# Identification of the first plant caffeoyl-quinate esterases in *Cichorium intybus*

**DOI:** 10.3389/fpls.2025.1632036

**Published:** 2025-08-20

**Authors:** Antoine Mallavergne, David Mathiron, Roland Molinié, Jean-Louis Hilbert, David Gagneul

**Affiliations:** ^1^ Joint Laboratory CHIC41H University of Lille-Florimond-Desprez, Université de Picardie Jules Verne, Université de Liège, Univ. Lille, Junia, UMRT 1158 BioEcoAgro - Specialized Metabolites of Plant Origin, Villeneuve d’Ascq, France; ^2^ BIOlogie des Plantes et Innovation (BIOPI), Université de Picardie Jules Verne, Université de Liège, Univ. Lille, Junia, UMRT 1158 BioEcoAgro - Specialized Metabolites of Plant Origin, Amiens, France; ^3^ Plateforme Analytique (PFA), Université de Picardie Jules Verne, Amiens, France

**Keywords:** chlorogenic acids, GDSL, chicory, caffeic acid, caffeoyl-putrescine, tobacco

## Abstract

Chlorogenic acid (5-CQA) is a caffeic acid ester widely accumulated in higher plants. It plays roles in defense against biotic and abiotic stresses. As its biosynthetic pathway shares common enzymes and intermediates with that of lignin, 5-CQA has long been hypothesized to be involved in lignin formation. However, to date, no plant enzymes have been identified that efficiently convert 5-CQA into lignin precursors. While investigating enzymes involved in the conversion of 5-CQA to isochlorogenic acid (3,5-DiCQA) in chicory (*Cichorium intybus*), we identified two enzymes from the GDSL esterase/lipase family, CiCQE1 and CiCQE3. Biochemical characterization and functional analysis in tobacco revealed that both enzymes can hydrolyze 5-CQA and 3,5-DiCQA to release caffeic acid (CA) both *in vitro* and *in planta*. The genes encoding CiCQE1 and CiCQE3 are predominantly expressed in chicory roots, where 5-CQA and 3,5-DiCQA accumulate to high levels. When transiently expressed in tobacco leaves, accumulation of caffeoyl-putrescine in addition to CA was observed. This may suggest that released CA may be converted to caffeoyl-CoA to fuel other metabolic paths. The hydrolysis of caffeoyl-shikimate, a compound structurally close to 5-CQA, to caffeic acid, and its subsequent conversion to caffeoyl-CoA, has been shown to be an important step in the biosynthesis of G and S monolignols. Since CiCQE1 and CiCQE3 catalyze similar reactions using 5-CQA as substrate, these enzymes may represent a novel route for 5-CQA remobilization in chicory roots. Further functional characterization of the role of these genes using mutant lines is still required to fully understand their role in planta.

## 1 Introduction

**Figure 1:**
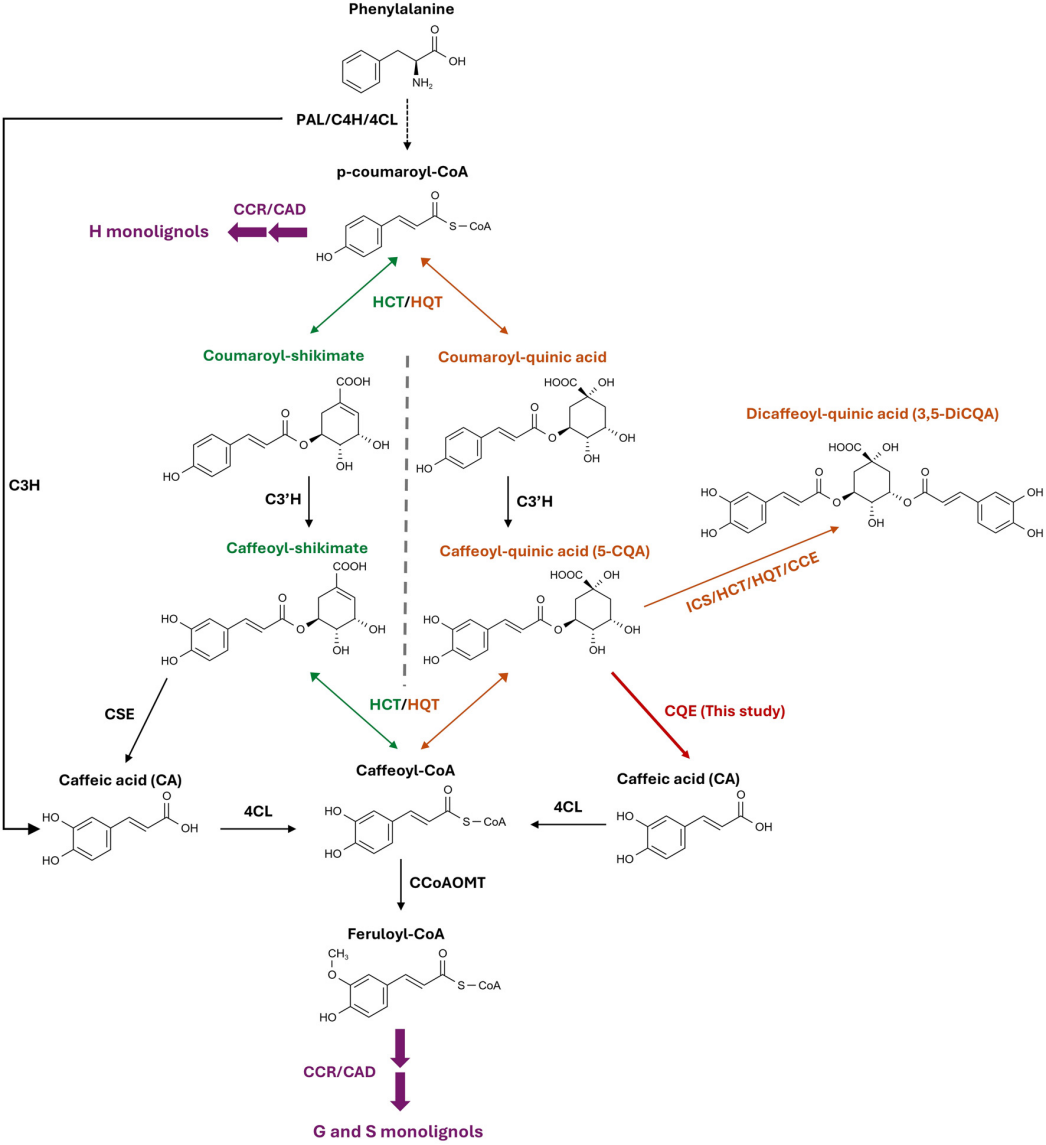
Caffeoyl-quinate and caffeoyl-shikimate biosynthetic pathways in land plants. Both compounds are derived from the core phenylpropanoid pathway generating p-coumaroyl-CoA. PAL, phenylalanine ammonia lyase; C4H, cinnamate 4-hydroxylase; 4CL, 4-coumaroyl-CoA ligase; CCR, cinnamoyl-CoA reductase; CAD, cinnamoyl alcohol dehydrogenase; HCT, hydroxycinnamoyl-CoA shikimate/quinate hydroxycinnamoyltransferase; HQT, hydroxycinnamoyl-CoA quinate/shikimate hydroxycinnamoyltransferase; C3H, p-coumarate 3-hydroxylase; C3’H, p-coumaroyl ester 3’-hydroxylase; CSE, caffeoyl-shikimate esterase; ICS, isochlorogenate synthase; CCE, chlorogenic acid condensing enzyme; CCoAOMT, caffeoyl-CoA O-methyltransferase.

Chlorogenic acid (caffeoyl-quinic acid, 5-CQA) and its derivative, isochlorogenic acid (dicaffeoyl-quinic acid, 3,5-DiCQA), are among the most abundant phenylpropanoids in higher plants including, but not limited to, species belonging to the *Asteraceae*, the *Solanaceae*, the *Rosaceae*, the *Lamiaceae* and the *Rubiaceae* (Clifford et al., 2017; Alcázar Magaña et al., 2021). Although their exact role in plants still remains unclear, their accumulation is strongly induced in response to various biotic and abiotic stresses, as well as upon activation by transcription factors such as MYB, suggesting their involvement in plant protection (Luo et al., 2008; Kundu and Vadassery, 2019; Bernard et al., 2020). 5-CQA and 3,5-DiCQA are formed by combining quinic acid with one or two caffeoyl moieties, respectively. Due to their high number of conjugated double bonds and hydroxyl groups, these compounds have high reactive oxygen species (ROS) scavenging activities *in vitro* and have been shown to protect plants from UV-B radiation (Islam et al., 2003; Niggeweg et al., 2004; D’Orso et al., 2023). 5-CQA has also been shown to inhibit the growth of fungi, bacteria and several herbivores, making it an important mediator of plant chemical defense (Ikonen et al., 2001; Leiss et al., 2009; Lee et al., 2017). 5-CQA is derived from p-coumaroyl-CoA, a key intermediate in the core phenylpropanoid pathway (**Figure 1**). Two BAHD acyltransferases, hydroxycinnamoyl-CoA quinate/shikimate hydroxycinnamoyltransferase (HQT) and hydroxycinnamoyl-CoA shikimate/quinate hydroxycinnamoyltransferase (HCT), catalyze the formation of p-coumaroyl-quinate and p-coumaroyl-shikimate through the transfer of p-coumaroyl-CoA to quinate and shikimate, respectively (Hoffmann et al., 2003). While HQT prefers quinate and HCT shikimate, they can use both substrates to form the corresponding p-coumaroyl conjugate (Lallemand et al., 2012). These two esters are then hydroxylated by cinnamate 3’-hydroxylase (C3’H), leading to the synthesis of caffeoyl-shikimate and 5-CQA, the two core molecules of this pathway (Schoch et al., 2001). The conversion of caffeoyl-shikimate to caffeoyl-CoA has been identified as an important step in the biosynthesis of G and S lignin monomers (Vogt, 2010). Although both HCT and HQT have been shown to catalyze the formation of caffeoyl-CoA from caffeoyl-shikimate and 5-CQA *in vitro*, their catalytic efficiency is too low to support a major role *in planta* (Hoffmann et al., 2003; Niggeweg et al., 2004; Legrand et al., 2016). The identification of a caffeoyl-shikimate esterase (CSE), which hydrolyses caffeoyl-shikimate to release caffeic acid (CA), has shed light on an alternative pathway for caffeoyl-CoA production (Vanholme et al., 2013) (**Figure 1**). The essential role of CSE in lignin biosynthesis has since been demonstrated *in vivo* in *Arabidopsis thaliana*, *Medicago truncatula*, and *Populus tremula x alba*, where *CSE* knockout mutants display severe growth defects along with an enrichment in H-type monolignols (Ha et al., 2016; Saleme et al., 2017). However, the absence of CSE homologs in some lignified plant species and the mitigated phenotype observed in poplar *CSE*-silenced lines suggest the existence of other mechanisms that control caffeoyl-CoA formation (Saleme et al., 2017). For instance, in switchgrass (*Panicum virgatum*), a bifunctional ascorbate peroxidase, the coumarate 3-hydroxylase (C3H), has been shown to directly convert p-coumarate to CA, bypassing the shikimate pathway entirely (Barros et al., 2019).

Since the biosynthesis of 5-CQA and lignin share common precursors and enzymes, it has long been hypothesized that 5-CQA serves as an intermediate in lignin formation (Silva et al., 2019). However, despite the observed correlations between 5-CQA accumulation and lignification in several plant species, there is no direct evidence to support the involvement of 5-CQA in lignin synthesis (Díaz et al., 1997; Joët et al., 2009; Garrett et al., 2016). For example, silencing *HQT* in tobacco significantly reduces 5-CQA levels without affecting lignin content or plant phenotype under standard growth conditions (Cardenas et al., 2021). However, 5-CQA may act as a storage form of CA that can be remobilized under specific environmental factors (Silva et al., 2019). Since CSE has not yet been shown to be able to hydrolyze 5-CQA, other enzymatic mechanisms leading to its breakdown may exist in plants to support this hypothesis (Escamilla-Treviño et al., 2014). 3,5-DiCQA is formed in plants by the transfer of the caffeoyl moiety of one 5-CQA to another 5-CQA (**Figure 1**). This reaction has been shown to be catalyzed by enzymes belonging to three different families of acyltransferases: HCT and HQT, two BAHDs, the isochlorogenate synthase (ICS), a GDSL and the chlorogenic acid condensing enzyme (CCE), a SCPL (Lallemand et al., 2012; Moglia et al., 2014; Miguel et al., 2020; Huang et al., 2024). As it is often accumulated in large amounts together with 5-CQA, 3,5-DiCQA could also serve as a source of CA in the caffeic acid ester metabolism. Chicory (*Cichorium intybus* L.) is an *Asteraceae* that accumulates an interesting pattern of caffeic acid esters (Legrand et al., 2016). While 5-CQA is found in all parts of the plant, 3,5-DiCQA accumulates almost exclusively in the roots, whereas high levels of the caffeoyl-tartaric acid esters, caffeoyl-tartaric (CTA) and dicaffeoyl-tartaric acid (DiCTA), are found specifically in leaves. Chicory is therefore a good model to study the metabolic pathway of these high-value bioactive compounds considering that the metabolism of these molecules is tightly interconnected.

Here, two chicory enzymes from the GDSL family, the caffeoyl-quinate esterases CiCQE1 and CiCQE3, were identified as efficient chlorogenate esterases by *in vitro* recombinant protein characterization and transient expression in *Nicotiana benthamiana* leaves. This newly described hydrolase activity in plant releases CA from 5-CQA and 3,5-DiCQA both *in vitro* and *in planta*. In tobacco expressing *CiCQEs*, the accumulation of CA, together with caffeoyl-putrescine, confirms the remobilization of CA toward caffeoyl-CoA, which will, in turn, fuel other biosynthetic pathways. Finally, qPCR analysis in chicory showed that *CiCQE1* and *CiCQE3* are predominantly expressed in roots, where 5-CQA and 3,5-DiCQA are located, suggesting a possible *in planta* role in their hydrolysis and remobilization.

## 2 Materials and methods

### 2.1 Plant material and growth conditions

Seeds of *C. intybus* L. var. Orchies were provided by Florimond-Desprez (Cappelle en pévèle, France). Following germination, seedlings were cultivated hydroponically on 0.5 X Murashige and Skoog (MS) medium (Murashige and Skoog, 1962) in a greenhouse for 6 weeks (16/8-h light/dark cycle; 24/18°C light/dark phase). Samples for cDNA synthesis and qRT-PCR experiments were collected in biological triplicates on a weekly basis, starting from week 3. Each plant was divided into three parts: root, young leaves and old leaves. All samples were immediately snap frozen in liquid nitrogen and stored at -80°C until needed. *N. benthamiana* were grown on soil under identical greenhouse conditions (16/8 h light/dark cycle; 24/18°C light/dark phase).

### 2.2 Extraction and analysis of polyphenols

Methanolic extractions were performed identically for *C. intybus* and *N. benthamiana*. Lyophilized plant material was powdered using a ball mill and 20 mg were resuspended in 1 mL of a methanol/water/acetic acid mixture (75/23/2, v/v/v). The mixtures were then incubated for 1 h at 4°C under agitation in the dark. Homogenates were then clarified by centrifugation at 14,000 *g* for 15 min at 4°C, and the supernatants were filtered through a 0.45 µm filter. The extracts were analysed by HPLC-DAD and UPLC-MS-MS as previously described (Bernard et al., 2022). For the calculation of the kinetic parameters, another gradient was developed in HPLC-DAD to achieve a better separation of 5-CQA and CA. The mobile phase was composed of water (as solvent A) and acetonitrile (as solvent B) both acidified with 0.1% ortho-phosphoric acid. The solvents were delivered at a flow rate of 1.1 mL min ^-1^. Oven temperature was set to 45°C. The following gradient of solvent B was used: 5-13% (0-1 min), 13-15% (1-11 min), 15-70% (11-12 min), 70% (12-13 min), 70-5% (13-14 min), 5% (14-21 min). For visualization and quantification, wavelength was set at 320 nm. All compounds were identified by comparison of their retention times, UV and mass spectra with those of commercial standards. Quantification was performed by reference to external standard calibration curves for each compound.

### 2.3 UHPLC-ESI-HRMS analysis

UHPLC-ESI-HRMS analysis was carried out on an ACQUITY UPLC I-class chain coupled to the Vion IMS Quadrupole time-of-flight high-resolution mass spectrometer (HRMS), equipped with an electrospray ionization (ESI) source (Z-spray) and an additional spray for the reference compound (Waters, Manchester, UK). A double detection was performed using a photodiode array (PDA) detector (UV detection between 210 and 400 nm) and by ESI-HRMS. One µL of the sample was injected into a Kinetex Biphenyl column (2.1 x 100 mm; 1.7 μm) (Phenomenex, Torrance, California, USA) heated to 55°C. Elution was performed with a mobile phase flow rate of 0.5 mL min^-1^ consisting of a mixture of water (A) and methanol (B) containing 0.1% of formic acid. The gradient starts at t = 0, ratio (A/B) 80:20; at t = 0.5 min, 80:20; at t = 5 min, 40:60; at t = 6 min, 10:90; at t = 7 min, 10:90; at t = 7.5 min, 80:20; and at t = 10 min, 80:20. ESI-HRMS analysis was performed in positive ionization mode over the mass range 50–1000 Da with ESI source parameters as follows: capillary voltage 3 kV, sampling cone voltage 40 V. Data were acquired in high-definition MS^E^ (HDMS^E^), a data independent analysis (DIA) mode using drift-time filtering, which consists of alternating a Low-Energy function (CE 6 eV) as well as a second High-Energy function (CE 20 to 50 eV) to obtain information on fragment ions. Data acquisition and processing was performed with UNIFI software (version 1.9.4, Waters).

### 2.4 BLAST search and sequence analysis

Homologous searches in chicory were conducted using the Basic Local Alignment Search Tool (BLAST). The IbICS protein sequence (GenBank accession QOH99319) was used as a query in a tBlastn search against the published industrial chicory genome (Waegneer et al., 2023). Signal peptide prediction was carried out using TargetP 2.0 (https://services.healthtech.dtu.dk/services/TargetP-2.0/), SignalP 5.0 (https://services.healthtech.dtu.dk/services/SignalP-5.0/) and Deeploc 2.0 (https://services.healthtech.dtu.dk/services/DeepLoc-2.0/). The three programs gave the same prediction. The molecular weight and isoelectric point of the predicted proteins were obtained from the ExPASy compute pI/MW tool (https://web.expasy.org/compute_pi/). The nucleotide sequences of candidate genes were translated, and multiple sequence alignment was performed using Muscle algorithm (Edgar, 2004) in MEGA 11 software (Tamura et al., 2021) to identify conserved domains and pairwise similarity. Amino acid conservation and alignment were visualized using Jalview 2.11.2.6 (Waterhouse et al., 2009). Phylogenetic tree construction was based on the multiple sequence alignment using the neighbour-joining method (Saitou and Nei, 1987) with 1000 bootstrap replicates in MEGA 11 software.

### 2.5 RNA extraction and cDNA synthesis

Plant samples stored at -80°C were ground in liquid nitrogen using a mortar and pestle. Approximately 100 mg of powdered tissue was used for extraction. Total RNA was isolated using the NucleoSpin RNA Plus Kit (Macherey-Nagel). Prior to cDNA synthesis, complete removal of contaminating genomic DNA was performed using the Turbo DNA-free™ kit (Invitrogen) by doing a routine DNAse treatment on 10µg of purified RNA samples. RNA quality and concentration were assessed on an Agilent 2100 Bioanalyzer using the 2100 Expert software. For candidate gene amplification and subsequent cloning, RNA samples from different tissues (roots, young/old leaves) and developmental stages (3 to 6 weeks) were pooled. For qRT-PCR experiments, RNA was extracted from different parts of 3-week-old chicory plants. cDNA synthesis was performed using 1 µg total RNA with the SuperScript IV Reverse Transcriptase Kit (Invitrogen) and oligo (dT)_20_.

### 2.6 qRT-PCR analysis

The *TIP41* gene was used as a reference for normalisation, based on previous experiments and assessment of its expression stability among samples used in this study (Delporte et al., 2015). *CiCQEs* and *TIP41* specific primers were designed using Primer 3 software (https://primer3.ut.ee/) with default parameters (Tm: 60 ± 1°C; 18-25 nucleotides; 40-60% GC; amplicon length from 60 to 150 bp) (**Supplementary Table S1**). The amplification efficiency of each primer set was determined to be 109.2, 110.3, 104.6 and 103.2% for *CiCQE1*, *CiCQE2*, *CiCQE3* and *TIP41*, respectively. qRT-PCR was performed as described in Delporte et al. using the iQ SYBR Green Supermix (Bio-Rad) in a final volume of 20 µL, containing 10 µL of iQ™ SYBR^®^ Green supermix (2x), 100 ng of DNA-free RNA and 5 µM of each primer (Delporte et al., 2015). The obtained cycle thresholds were normalized to *TIP41* values. The following program was used: initial denaturation at 95°C for 3 min followed by 40 cycles including 95°C for 10 s and 60°C for 30 s. Melting curves were generated for each sample to verify the amplification specificity. To control for genomic DNA contamination, reactions were also performed with non-retro-transcribed RNA. Relative gene expression levels were calculated according to the Pfaffl equation (Pfaffl, 2001).

### 2.7 Transient expression of CiCQEs in *N. benthamiana* leaves

Full-length sequences of CiCQE1 (Genbank accession PX093951), CiCQE2 (Genbank accession PX093952) and CiCQE3 (Genbank accession PX093953) without stop were amplified from a pool of cDNA (see above) with a proofreading polymerase (PrimeSTAR HS DNA polymerase, Takara). To minimise allelic variation, each amplification was performed in triplicate. The primers used are listed in **Supplementary Table S1**. For CiCQE2, cloning from gDNA or by removing the signal peptide was also performed. Using Gateway cloning™, entry vectors pDONR-Zeo_CiCQE1, pDONR-Zeo_CiCQE2, pDONR-Zeo_CiCQE3 were obtained and used to generate the expression vectors pEAQ-HT-DEST3_CiCQE1, pEAQ-HT-DEST3_CiCQE2, pEAQ-HT-DEST3_CiCQE3, respectively. All constructs were verified by Sanger sequencing. The pEAQ-HT-DEST3 vector adds a C-terminus 6x His tag to the protein and co-expresses the silencing inhibitor P19. The constructs pEAQ-HT-DEST3_CiCQEs were introduced into *Rhizobium radiobacter* strain LBA4404 (Invitrogen) by electroporation (V=2,500, µF=25, Resistance=400 Ω) and selected on YEB plates containing 25 µg mL^-1^ of rifampicin and 50 µg mL^-1^ of kanamycin. Four independent clones per construct were used to inoculate 20 mL of liquid YEB medium containing 50 µg mL^-1^ of kanamycin and grown overnight at 28°C with shaking at 210 rpm to an OD_600_ of 0.8-1. The bacterial cultures were then pelleted by centrifugation for 10 min at 3,000 *g* at 20°C and resuspended in infiltration buffer (10 mM MgCl_2_, 10 mM MES, 100 µM acetosyringone, pH 5.7) to a final OD_600_ of 1. After a 3-hour incubation at room temperature, the abaxial epidermis of 6-week-old *N. benthamiana* leaves was infiltrated with the bacterial suspension using a needless syringe. Plants infiltrated with agrobacteria carrying the empty pEAQ-HT-DEST3 vector served as negative controls.

### 2.8 Purification of recombinant CiCQEs

Transformed *N. benthamiana* leaves were harvested 3 days post-infiltration, immediately frozen in liquid nitrogen, and stored at -80°C until protein extraction. Frozen leaf tissue expressing the pEAQ-HT-DEST3_CQEs constructs was ground in liquid nitrogen using mortar and pestle. An appropriate volume of binding buffer (20 mM sodium phosphate, 0.5 mM NaCl, 20 mM imidazole, pH 7.4) was immediately added to the resulting powders (4 mL g^-1^ FW). Following a 15-minutes incubation on ice, the crude extracts were clarified by centrifugation for 10 min at 4,000 *g* at 4°C and passed through a 0.22 µm filter (Sarstedt). Flow-throughs were used immediately for protein purification.

His-tagged proteins were purified on 1 mL HisTrap HP columns (GE Healthcare) loaded with Ni^2+^, following supplier protocols. Briefly, the column was first equilibrated with 10 mL of binding buffer (20 mM sodium phosphate, 0.5 mM NaCl, 20 mM imidazole, pH 7.4) before loading with filtered protein extracts. Unbounded proteins were then washed out with 15 mL of binding buffer. Finally, bound His-tagged proteins were eluted in five 1 mL fractions with elution buffer (20 mM sodium phosphate, 0.5 mM NaCl, 500 mM imidazole, pH 7.4). The enriched protein fractions were pulled and desalted on a pD-10 desalting column (GE Healthcare). Proteins were eluted in storage buffer (10 mM phosphate buffer pH 7.2, 20% glycerol) and concentrated ten-fold on Vivaspin 6 columns (Sartorius) with a 30 kDa mass cut-off. Concentrated His-tagged proteins were stored at -20°C until further use.

### 2.9 Protein quantification, SDS-PAGE and immnuoblot analysis

Protein concentration was estimated using the Bradford reagent (Bio-Rad). A standard calibration curve was generated using BSA ranging from 0 to 1 mg mL^-1^. SDS-PAGE and immunoblot analyses were performed as described elsewhere. For Coomassie blue staining and Western blot analysis, 5 µg of purified proteins were loaded per lane. Proteins were transferred to a PVDF membrane (Trans-Blot Turbo Transfer Pack). Immunodetection was carried out using a mouse anti-penta-His antibody (1:1,200, Qiagen) as the primary antibody and a mouse anti-IgG antibody (1:2,500, Promega) as the secondary antibody. Revelation was performed using the NBT/BCIP Reagent (Bio-Rad).

### 2.10 *In vitro* assays of purified CiCQEs

Enzymatic assays were performed in a total volume of 15 µL of tri-buffer (0.1 M acetic acid, 0.1 M Tris, 20 mM MES, pH 6), containing 0.4 µg of purified recombinant his-tagged protein. For the evaluation of CiCQEs activity on chicory crude methanolic extracts, root and leaf extracts prepared as described were first dried using a SpeedVac concentrator and then resuspended in the same volume of tri-buffer. Standard assays were performed with 1 mM of substrates in the reaction mix and incubated for 1 h at 30°C. For kinetic analysis, varying concentrations of 5-CQA (10 µM to 10 mM) were used with 0.2 µg of CiCQEs, incubated for 15 min at 30°C based on preliminary experiments. The optimal pH was determined using 500 µM of 5-CQA in 15 µL of various buffers (0.1 M citrate, 0.1 M acetate, 0.1 M phosphate and 0.1 M tris) ranging from pH 3 to 9 containing 0.2 µg of purified CiCQEs. Reactions were run for 30 min at 30°C. All reactions were stopped by the addition of 45 µL of methanol, filtered through 0.45 µm filter plates (AcroprepTM PALL) and immediately analyzed by HPLC-DAD (Shimadzu). All reactions were performed in triplicate. Kinetic parameters were determined by fitting Michaelis-Menten curves using GraphPad Prism Software. Optimal pH was calculated using the same software by fitting a Gaussian curve to the data.

### 2.11 Esterase assays using *p*-nitrophenyl-acetate

Esterase activity was assayed in a 100 µL reaction volume containing tri-buffer (0.1 M acetic acid, 0.1 M Tris, 20 mM MES, pH 6), 1 mM of *p*-nitrophenyl-acetate and 0.2 µg of purified CiCQEs. In control reactions, purified proteins were replaced with storage buffer (10 mM phosphate buffer pH 7.2, 20% glycerol). Enzymatic reactions were incubated at 30°C for 15 min. Absorbance at 347 nm was immediately measured using a Thermo Scientific Multiskan Spectrum. The concentration of *p*-nitrophenol was determined from an external calibration curve generated with a commercial standard of *p*-nitrophenol.

### 2.12 Statistics

Statistical analysis was made on R 4.1.2. Depending on the data analyzed, ANOVA or Student’s t-test were performed.

## 3 Results

### 3.1 Identification of candidates involved in chlorogenic acid metabolism in chicory

**Figure 2:**
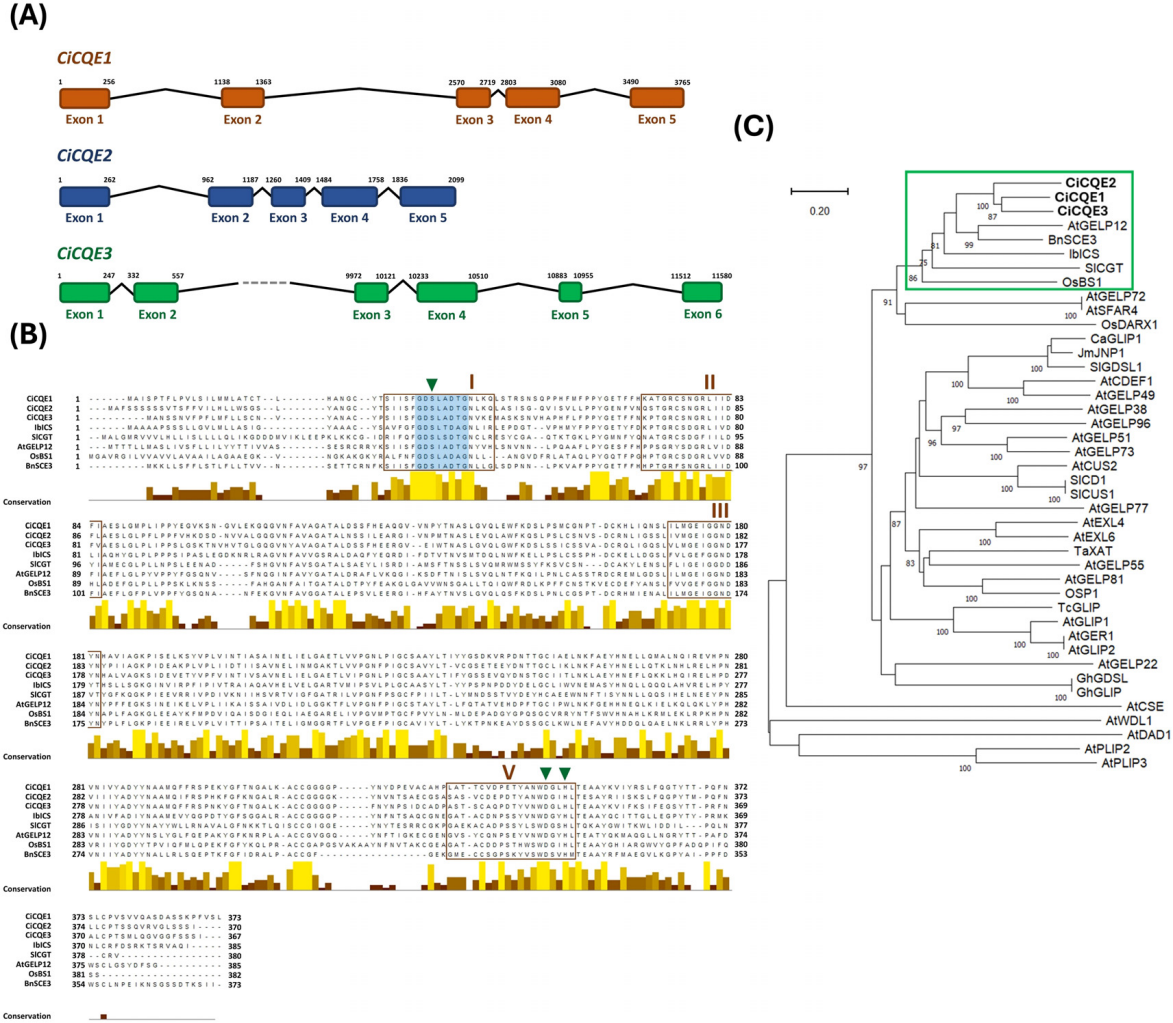
Sequence analysis of the three candidate genes of the GDSL family. **(A)** Schematic diagram of *CiCQE1*, *CiCQE2* and *CiCQE3* gene structures. Colored boxes represent exons and introns are shown as black lines. The grey dotted lines represent the 9 kb intron found in *CiCQE3*. **(B)** Alignment of the deduced amino acid sequence of CiCQEs with other biochemically characterized GDSL proteins. Alignment was performed using the Muscle algorithm. The conserved GXSXXDXG motif is highlighted in blue. Conserved blocks of the SGNH family are framed in orange (I, II, III and V). Amino acids of the catalytic triad are indicated by green triangles. Conservation between aligned sequences is shown in yellow. **(C)** Phylogenetic tree of CiCQEs and several characterized GDSL esterases/transferases. See **Supplementary Table S2** for details of protein sequences and accession numbers. The green frame marks the clade containing the CiCQEs and IbICS used as a query in our tblastn search. The length of the lines indicates the relative distance between nodes using the scale bar provided. Bootstrap was calculated with 1,000 replicates. Values higher than 75 are shown.

A GDSL enzyme that catalyzes the formation of 3,5-DiCQA from two molecules of 5-CQA was recently identified in sweet potato and named isochlorogenate synthase (IbICS) (Miguel et al., 2020). To identify homologous enzymes involved in 5-CQA metabolism in chicory, a tBLASTn search was conducted against the published industrial chicory genome (Waegneer et al., 2023) using the IbICS protein sequence as query. Among the multiple retrieved GDSL homologs, the three best hits were selected and hereafter named caffeoyl-quinate esterases i.e. *CiCQE1*, *CiCQE2*, *CiCQE3* for further analysis. Genomic sequence analysis revealed that *CiCQE1* and *CiCQE2* each consist of five exons and four introns, whereas *CiCQE3* contains an additional exon (**Figure 2A**). Their coding sequences are 1182, 1173 and 1164 bp long, respectively. In particular, *CiCQE3* has a large intron of 9 kb between exons 2 and 3. *CiCQE2* and *CiCQE3* are clustered on chromosome 6 together with several other predicted GDSLs, while *CiCQE1* is located on chromosome 5. The predicted lengths of the encoded proteins are 394, 391, and 388 amino acids (AA), respectively. Signal peptide prediction using SignalP, TargetP and DeepLoc softwares suggested N-terminal signal peptides in all three proteins, addressing them to the secretory pathway (**Supplementary Figure S1**). After removal of the signal peptides, the predicted protein molecular weights were 39.72 (CiCQE1), 38.91 (CiCQE2) and 39.19 (CiCQE3) kDa, with a isoelectric points (pI) of 5.15, 5.26 and 5.05, respectively. Pairwise sequence identity analysis revealed 64.14% similarity between CiCQE1 and CiCQE2, 63.71% between CiCQE2 and CiCQE3, and 69.29% between CiCQE1 and CiCQE3. All three candidate proteins contain the characteristic GXSXXDXG motif, which is conserved among members of the GDSL lipase/esterase family, as well as the four conserved blocks of the SGNH hydrolase family (**Figure 2B**). In addition, the catalytic triad Ser-Asp-His, which is essential for the esterase/transferase activity, is present in all three proteins, supporting their classification within the GDSL family. Phylogenetic analysis including several characterized GDSL proteins revealed that the three candidates cluster with IbICS and SlCGT (Teutschbein et al., 2010; Miguel et al., 2020), which are transferases using 5-CQA as a substrate as well as several GDSLs involved in the metabolism of cell wall components (Clauß et al., 2011; Zhang et al., 2017; Ursache et al., 2021) (**Figure 2C**). These results suggest a potential role for these chicory GDSLs in 5-CQA metabolism or related pathways.

### 3.2 Expression pattern of *CiCQEs* in different chicory tissues

**Figure 3:**
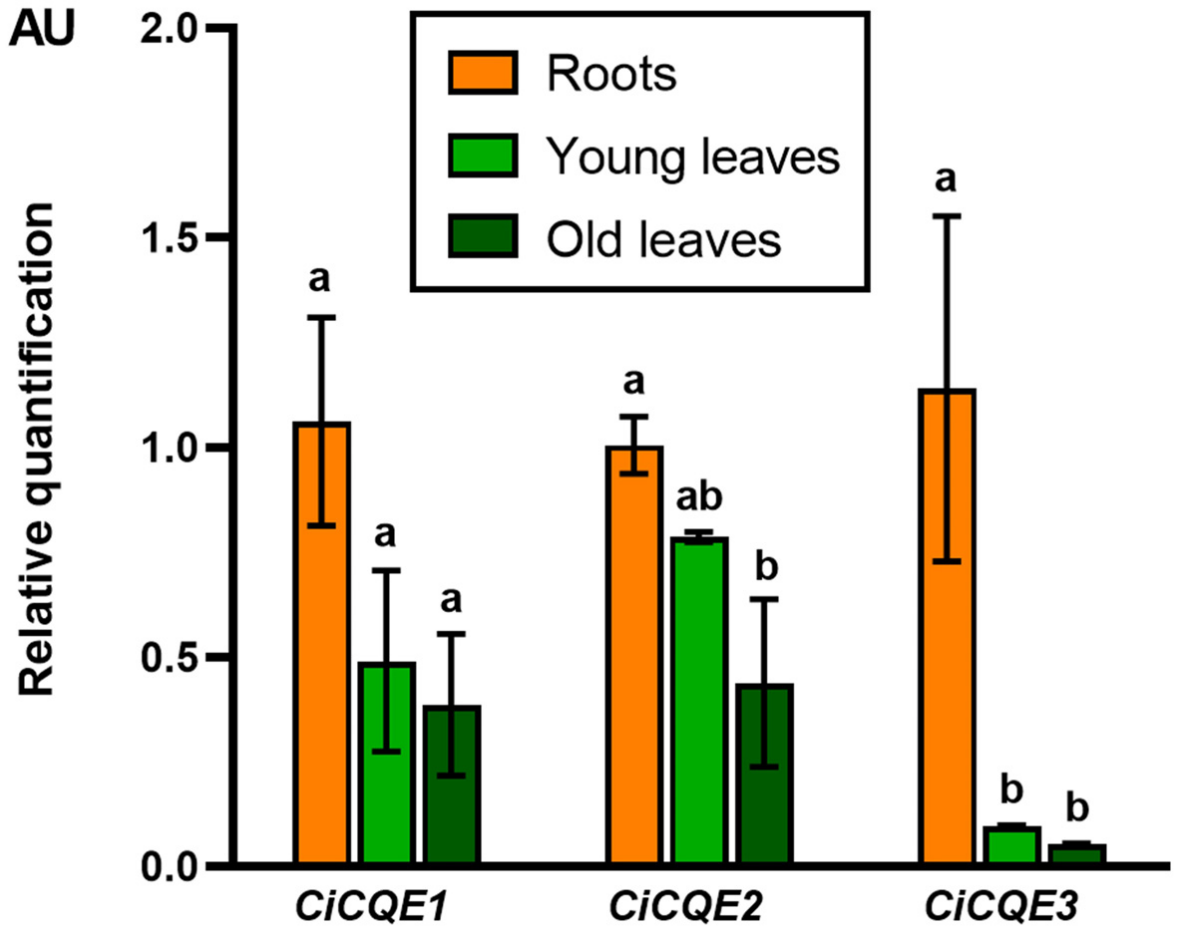
Expression profiles of *CiCQE1*, *CiCQE2* and *CiCQE3* in different tissues of chicory. Relative expressions of *CiCQEs* were determined by qRT-PCR on 3-week-old plants. Data were normalized to the expression of the reference gene *TIP41* and shown relative to the expression level measured in roots. Different letters indicate significant differences as determined by ANOVA (*P-value < 0.05*). Error bars indicate standard deviation (SD) calculated from three replicates. AU, Arbitrary unit.

To gain further insight into the potential roles of the candidates, their expression profiles were examined by quantitative real-time PCR (qRT-PCR). Given the differential accumulation of caffeoyl-quinic acids (5-CQA and 3,5-DiCQA) and caffeoyl-tartaric acids (CTA and DiCTA) between roots and leaves in chicory, expression analyses were conducted on both tissues, including leaves at different developmental stages. Transcript levels were normalized using *TIP41*, which was identified as the most stable reference gene among the samples tested. All three *CiCQEs* displayed higher expression levels in roots compared to leaves, although this difference was not statistically significant for *CiCQE1* (**Figure 3**). In particular, *CiCQE3* showed a tenfold higher expression in roots than in leaves. As 5-CQA and 3,5-DiCQA are the only caffeic acid esters accumulated in roots, these expression patterns suggest that the *CiCQEs* candidates are more likely to be involved in the metabolism of these compounds rather than caffeoyl-tartaric acid esters (Legrand et al., 2016). However, except for *CiCQE3*, the high expression of *CiCQE1* and *CiCQE3* in leaves did not support their role in 3,5-DiCQA synthesis as this compound is almost completely absent in leaves.

### 3.3 Production, purification and *in vitro* assays of recombinant CiCQEs

**Figure 4:**
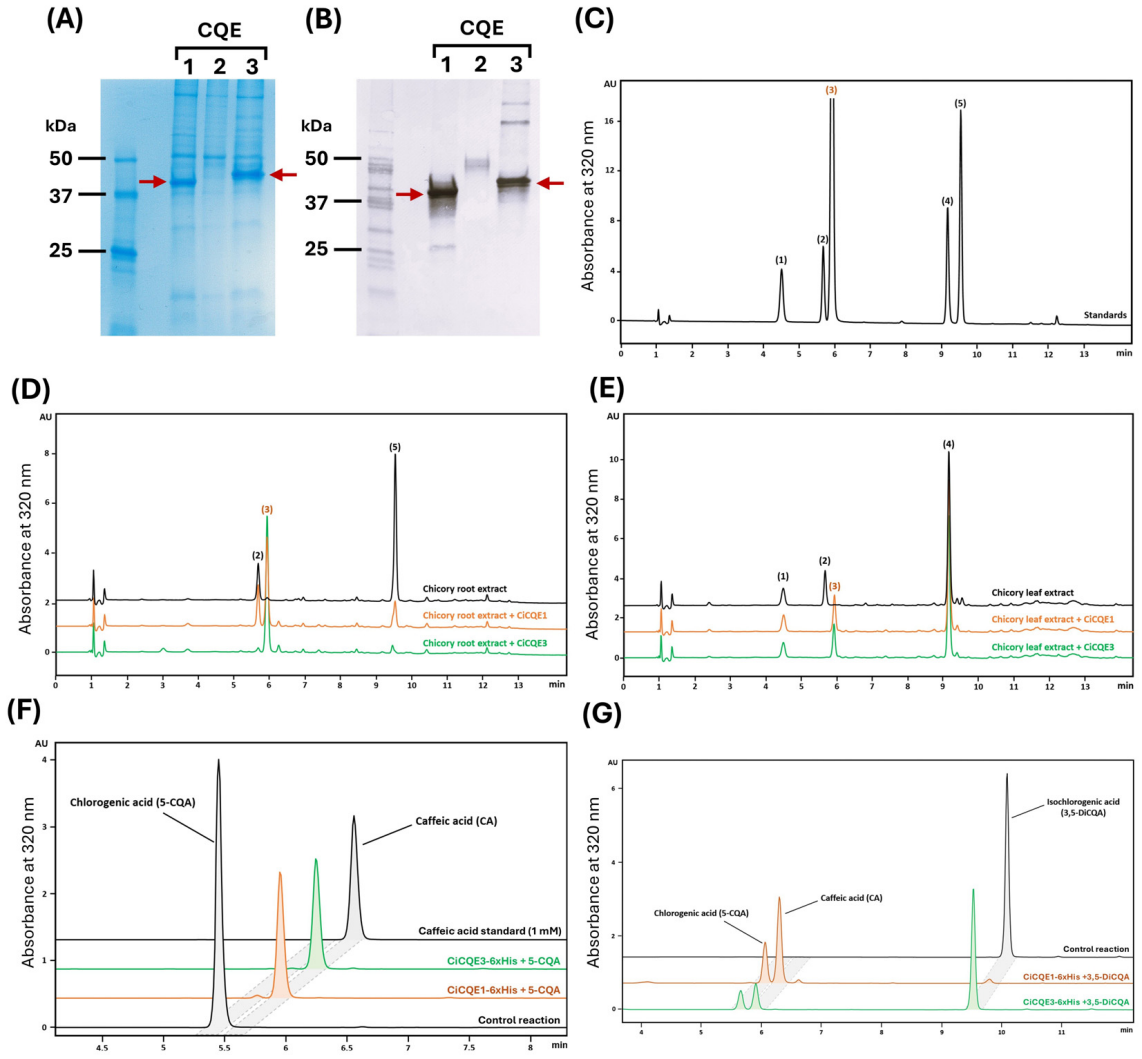
Purification and biochemical characterization of CiCQEs. **(A)** SDS-PAGE separation of the three proteins. The gel was stained with Coomassie blue. **(B)** Immunoblot analysis of the recombinant proteins confirming the purification of CiCQE1 and CiCQE3. A mouse anti-penta-his antibody (Qiagen) was used to label his-tagged proteins. The secondary antibody used was a mouse anti-IgG antibody (Promega). M corresponds to the size marker Precision Plus Protein TM All Blue Prestained Protein Standards. Red arrows indicate bands at the **(D, E)** HPLC chromatograms of in vitro assays of purified CiCQE1 (orange line) and CiCQE3 (green line) with chicory root **(D)** or leaf **(E)** methanolic extracts. The chromatogram of the native leaf and root extracts are depicted in black. The identity of caffeic acid was confirmed by mass spectrometry. **(F, G)** HPLC chromatograms of in vitro assays of purified recombinant proteins in the presence of 5-CQA **(F)** and 3,5-DiCQA **(G)**. The reactions were performed in tri-buffer pH 6.5 containing 1 mM of substrate. All reactions were carried out for 1 h at 30°C with 1 µg of purified CiCQE1 (orange line) or CiCQE3 (green line). (1) CTA, (2) 5-CQA, (3) CA, (4) diCTA, (5) 3,5-DiCQA. AU, Arbitrary unit.

To evaluate the enzymatic functions of CiCQEs, their corresponding genes were cloned and expressed in the plant heterologous system *N. benthamiana*. This system was chosen for its ability to perform appropriate post-translational modifications that are often lacking in *Escherichia coli*, resulting in inactive GDSL proteins (Teutschbein et al., 2010; Tan et al., 2014; Miguel et al., 2020). The *CiCQE1*, *CiCQE2* and *CiCQE3* coding sequences were amplified from chicory cDNA and fused to a C-terminal 6xHis tag for purification. N-terminal His-tag constructs were not generated, as each gene contains a predicted signal peptide at the N-terminus. Following transient expression in *N. benthamiana* leaves via *Rhizobium*-mediated transformation, proteins were extracted and purified on His-trap columns, based on histidine affinity to nickel. The presence and identity of CiCQE1 (39.72 kDa) and CiCQE3 (39.19 kDa) were confirmed by Coomassie blue staining and immunoblot analysis (**Figures 4A**, **B**). CiCQE2 did not give bands at the expected size despite multiple attempts of production and purification. To overcome potential problems of transcript processing or protein targeting, cloning from gDNA and of the truncated cDNA (without signal peptide sequence) were done and the resulting expression vectors were used to infiltrate tobacco. Unfortunately, no protein or activity could be detected in these conditions. Modeling and docking experiments performed with CiCQEs revealed that, despite sharing a similar 3D structure, 5-CQA might be bind in reverse position within the active site of CiCQE2, blocking the catalytic access of serine to the ester bonds (**Supplementary Figure S2**). This candidate was therefore not considered for further analysis. To assess enzymatic activity *in vitro*, purified CiCQE1-6xHis and CiCQE3-6xHis proteins were incubated with methanolic extracts of chicory leaves or roots (**Figures 4C**-**E**). Incubation with both enzymes did not result in the formation of 3,5-DiCQA, as was initially expected on the basis of sequence homology with IbICS. Instead, both enzymes surprisingly led to the accumulation of (CA) together with a strong reduction in the levels of 5-CQA and 3,5-DiCQA. Similarly, the incubation of purified CQEs with standards of 5-CQA and 3,5-DiCQA resulted in substrate degradation and accumulation of CA, as verified by mass spectrometry (m/z of 181 in positive mode) (**Figures 4F**, **G**) (**Supplementary Figures S3**, **S4**). Both enzymes were unable to metabolize caffeoyl-tartaric acids but showed hydrolytic activities toward caffeoyl-shikimate (data not shown). Incubations made with other isomers of 5-CQA and 3,5-DiCQA showed that CiCQE1 acts specifically on 5-CQA and 3,5-DiCQA, while CiCQE3 uses 5-CQA, 3,5-DiCQA and 1,5-DiCQA (**Supplementary Figure S5**). Collectively, these results suggest that CiCQE1 and CiCQE3 function as esterases rather than transferases, efficiently hydrolyzing 5-CQA and 3,5-DiCQA to release CA *in vitro*.

### 3.4 Measurement of CiCQE1 and CiCQE3 hydrolytic activity using *p*-nitrophenyl-acetate

**Figure 5:**
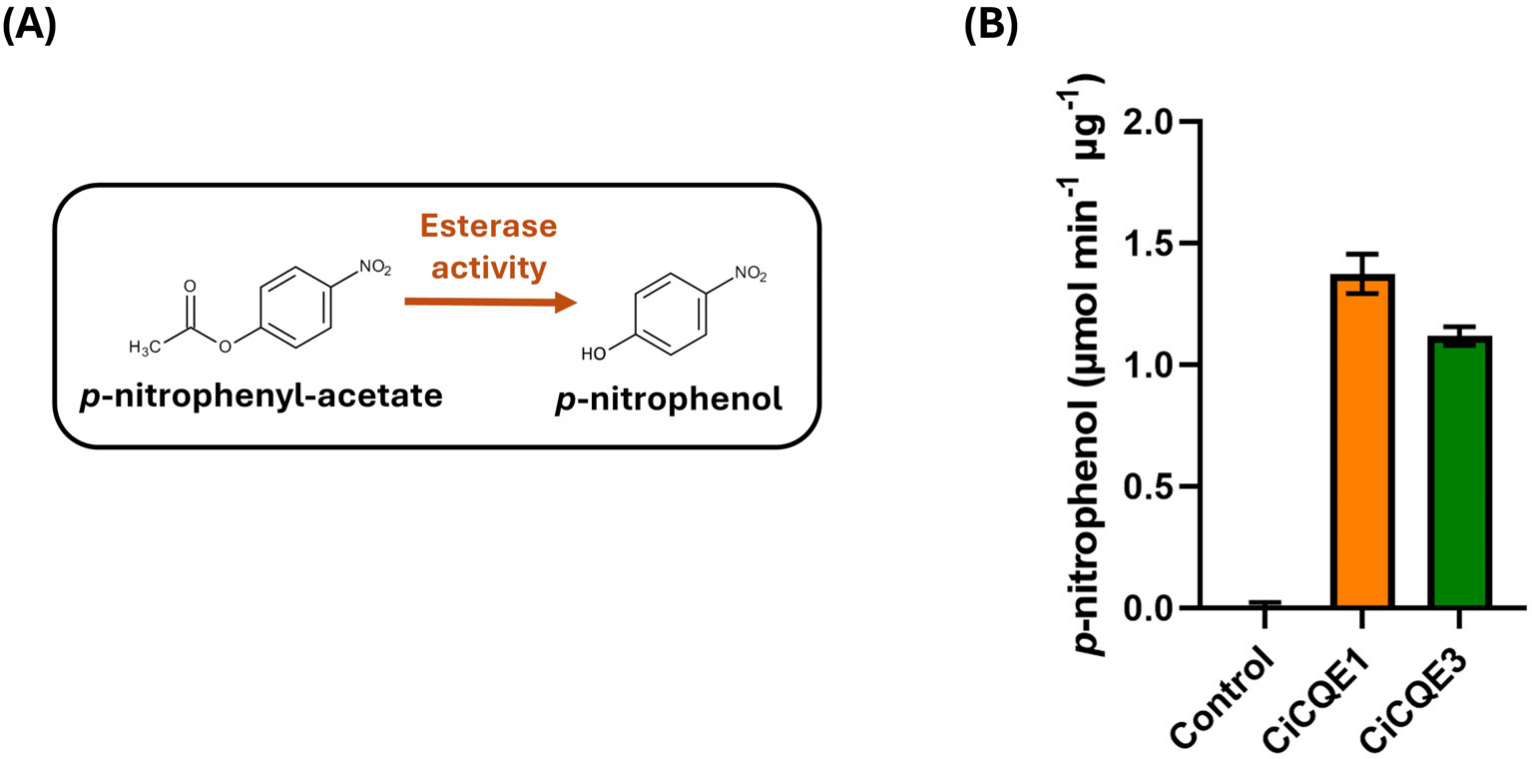
Esterase activity of CiCQE1 and CiCQE3 using *p*-nitrophenyl-acetate as substrate. **(A)** Hydrolysis of *p*-nitrophenyl-acetate by esterase induces the release of *p*-nitrophenol. **(B)** Velocity of the esterase activity of CiCQE1 and 2 in the presence of p-nitrophenol. Absorbance was monitored at 347nm. Error bars indicate standard deviation (SD) calculated from three replicates.

Previous data have shown that the two enzymes under investigation may most probably act as esterases. To test this, we monitored the esterase activity of both enzymes using *p*-nitrophenyl-acetate, a substrate commonly used to assess hydrolytic activities (Peng et al., 2016b). Upon hydrolysis, *p*-nitrophenyl-acetate releases *p*-nitrophenol, which has a stable absorbance at 347 nm, independent of pH, allowing for accurate quantification (**Figure 5A**). Purified CiCQE1 and CiCQE3 were then incubated with *p*-nitrophenyl-acetate after which the absorbance at 347 nm was measured. Both enzymes showed a significant accumulation of *p*-nitrophenol compared to the negative control (**Figure 5B**). CiCQE1 and CiCQE3 exhibit esterase activity, producing 1.38 and 1.12 µmol min^-1^ mg^-1^ enzyme of *p*-nitrophenol using *p*-nitrophenyl-acetate as substrate according to a calibration curve of *p*-nitrophenol. This analysis confirms that the candidate CiCQEs possess esterase activity, confirming their role as chlorogenate esterases *in vitro*.

### 3.5 Kinetic parameters of CiCQE1 and CiCQE3

**Figure 6:**
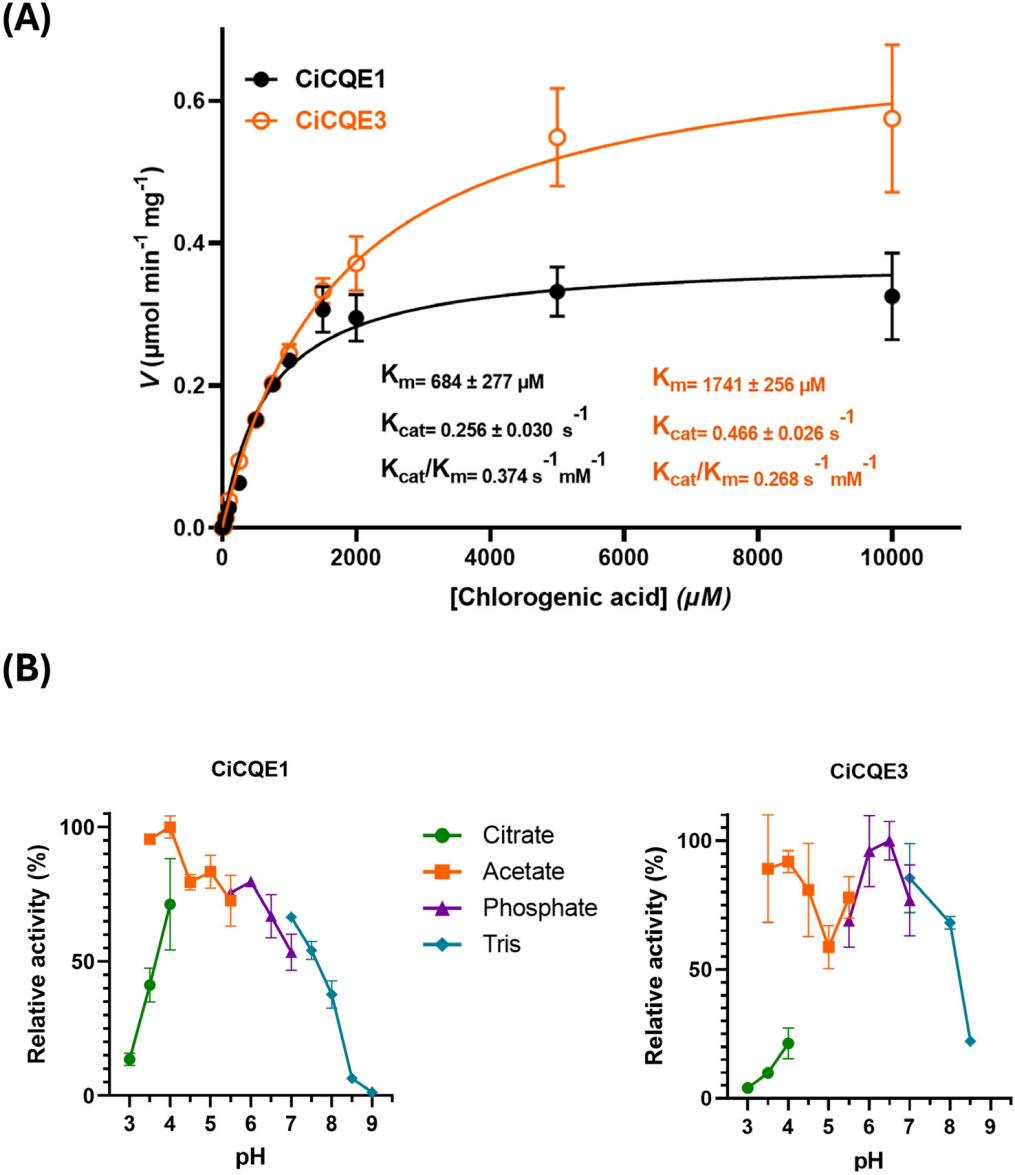
Kinetic parameters of CiCQE1 and CiCQE3. **(A)** Michaelis-Menten plot of CiCQE1 in black and CiCQE3 in orange toward 5-CQA hydrolysis. Enzymevelocity is given as μmol.min^-1^.mg^-1^ enzymes. The calculated values for K_m_, the turnover number K_cat_ and the K_cat/Km_ ratio are shown on the graph. **(B)** Optimal pH of both enzymes calculated using different buffers depending on the pH range. The results are shown as relative to the optimal pH. All experiments were performed in triplicates and the error bars indicate the standard deviation.

To further investigate the enzymatic activity of CiCQE1 and CiCQE3, we determined their kinetic parameters *in vitro* using 5-CQA as substrate (**Figure 6A**). The Michaelis-Menten Km values for CiCQE1 were determined to be 0.684 mM, while the turnover Kcat was estimated to be 0.256 s^-1^. In contrast, CiCQE3 exhibited a higher Km value of 1.740 mM, indicating a lower substrate affinity for 5-CQA. However, its Kcat was found to be 0.466 s^-1^, suggesting a higher catalytic rate. Despite this, both enzymes display relatively high Km values, indicating a low affinity to 5-CQA under *in vitro* conditions. Nevertheless, given the relatively high endogenous concentration of 5-CQA, around 4 µmol g^-1^ DW in chicory roots (Legrand et al., 2016), these compounds might be used *in vivo* as well. The Vmax values for CiCQE1 and CiCQE3 were comparable to those reported for AtCSE, when tested with caffeoyl-shikimate, which have significative activity *in planta* (Vanholme et al., 2013). The optimal pHs of CiCQE1 and CiCQE3 were then determined using different buffers depending on the pH range (**Figure 6B**). Both enzymes showed peak activity around pH 6. However, an increase in activity was observed at pH 4 in acetate buffer. These pH optima are consistent with the signal peptides of CiCQEs, which are predicted to direct proteins through the secretory pathway, either the vacuole or the apoplast. Both of this compartment are acidic cellular environments.

In addition to their activity on 5-CQA, both CiCQE1 and CiCQE3 were found to have hydrolytic activity on 3,5-DiCQA. However, the enzymatic hydrolysis of 3,5-DiCQA yields 5-CQA, which is also a substrate for CiCQEs, further hydrolyzed to CA (**Figure 4G**; **Supplementary Figure S5**). This sequential reaction hampers kinetic analysis, as it introduced overlapping substrate-product dynamics and prevented accurate determination of kinetic parameters for the initial hydrolysis of 3,5-DiCQA. Consequently, although both enzymes clearly hydrolyze 3,5-DiCQA, the catalytic efficiency toward this substrate could not be determined.

### 3.6 Functional analysis of CiCQEs in *N. benthamiana* leaves

**Figure 7:**
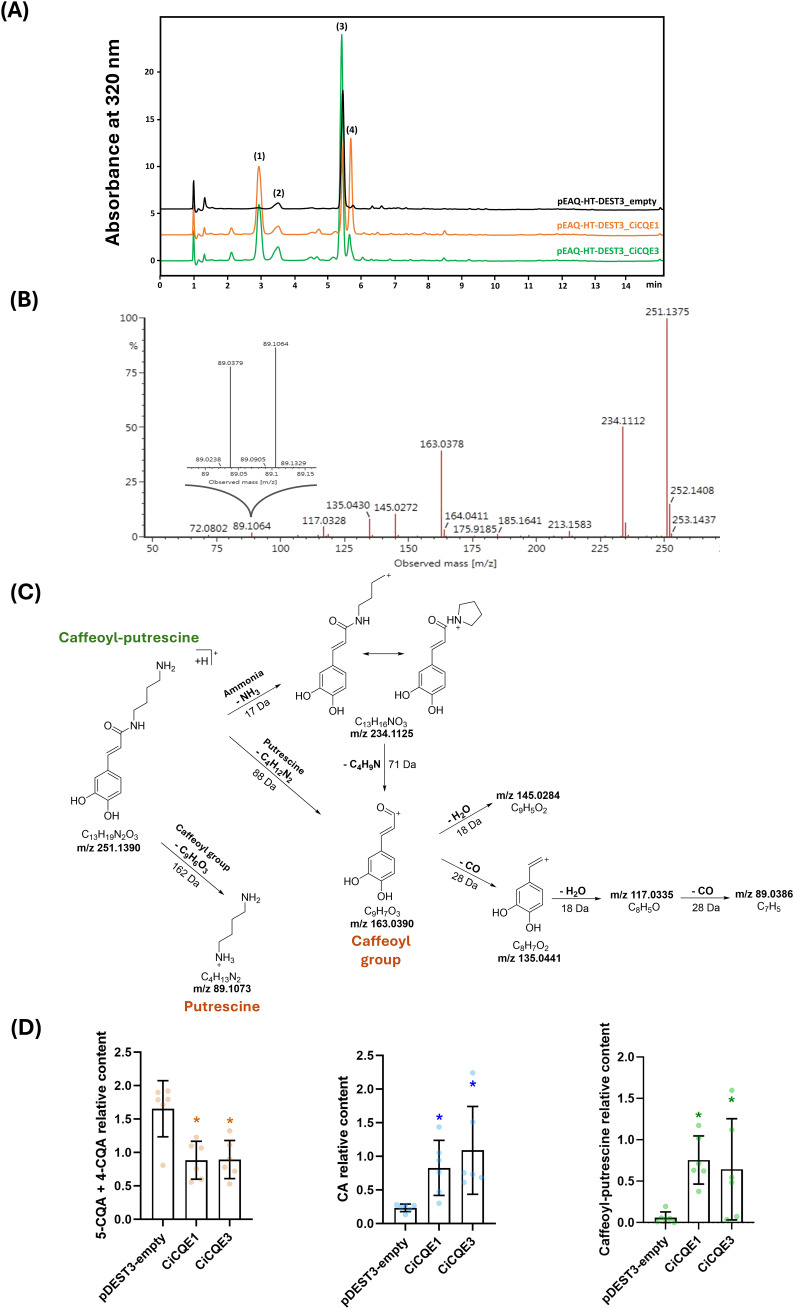
Analysis of phenolic profiles of Nicotiana Benthamiana transiently transformed leaves. **(A)** Chromatograms of N. benthamiana leaves transformed with the empty vector, pEAQ-HT-DEST3_CiCQE1 or pEAQ-HT-DEST3_CiCQE3 constructs. (1) Caffeoyl-putrescine, (2) 3-CQA, (3) 5-CQA + 4-CQA, (4) CA. **(B)** ESI-HDMS^E^ spectra (collision energy: Low Energy function 6 eV) of compound at RT 2.89 identified as caffeoyl-putrescine. **(C)** Proposed fragmentation scheme for caffeoyl-putrescine obtained according to ESI- HDMS^E^ data. **(D)** Relative levels of identified compounds in transformed leaves. The asterisks indicate significant differences as determined by t-test (*P*-value < 0.05) compared to the empty vector control. Experiments were run with six independent tobacco plants as replicates.

Tobacco leaves are known to accumulate high levels of 5-CQA. To investigate the functional role of CiCQE1 and CiCQE3 *in planta*, we analyzed the impact of their overexpression on the phenolic contents of *N. benthamiana* leaves. Methanolic extracts were prepared from leaves six days after agroinfiltration with constructs harboring either *CiCQE1* or *CiCQE3*. Leaves infiltrated with an empty vector served as a negative control. Infiltration with the two constructs resulted in significant accumulation of two compounds not detected in the negative control (**Figure 7A**). One of these was identified as caffeic acid (CA), in agreement with the *in vitro* characterization. The second compound was purified and analyzed by UPLC/ESI- HDMS^E^. A peak absent in the control was detected at retention time 2.89 min. It gave a [M+H]^+^ ion at mass-to-charge ration (m/z) of 251.1375 (**Figure 7B**). The elemental composition was determined to be C_13_H_19_N_2_O_3_. The HDMS^E^ spectra revealed fragment ions at m/z 234.1112, 163.0378 and 89.1064 which are specific of the fragmentation pattern of caffeoyl-putrescine (**Figure 7C**) (Baumert et al., 2001; Zhong et al., 2024). It should be noted that many fragment ions are present in Low Energy spectrum due to spontaneous fragmentation in the ESI source and most of them are found in the High Energy spectrum, differing only in their intensity. Based on the fragmentation pattern (**Figure 7C**), the fragment ion at m/z 234.1112 corresponds to the loss of NH_3_ (17 Da) from the precursor ion 251.1375, i.e. a NH_3_ loss from the terminal amine of putrescine part of caffeoyl-putrescine. The fragment at m/z 163.0378 corresponds to the caffeoyl and can be explained by loss of putrescine (88 Da) from caffeoyl-putrescine [M+H]^+^ ion at m/z 251.1375. The very low intensity fragment at 89.1064 observed in the Low Energy spectrum (**Figure 7B**) is diagnostic and corresponds to [M+H]^+^ of putrescine. The other fragments at m/z 145.0272, 135.0430, 117.0328 and 89.0379 came from the caffeoyl corresponding ion at m/z 163.0378. To conclude, HDMS^E^ fragmentation data are consistent with caffeoyl-putrescine as the second compound accumulated in *N. benthamiana* leaves. Caffeoyl-putrescine is a phenolamide synthesized from caffeoyl-CoA suggesting the formation of this intermediate in response to the overaccumulation of CA in transformed tobacco (Peng et al., 2016a; Cao et al., 2024). The accumulation of CA and caffeoyl-putrescine was consistent across biological replicates and correlated with a significant reduction in 5-CQA levels (**Figure 7D**). Although neochlorogenic acid (4-CQA), which is present in substantial amount in tobacco leaves, coelutes with 5-CQA in our experiments. Neither CiCQE1 nor CiCQE3 can use this isomer *in vitro*, as demonstrated in **Supplementary Figure S5**. The observed reduction in chlorogenic acids level in tobacco is therefore only due to the consumption of 5-CQA. Regarding neochlorogenic acid (3-CQA), tobacco leaves also accumulated this compound, however, no decrease in its accumulation was observed following the expression of *CiCQEs* in accordance with *in vitro* experiments. These results in tobacco support a role for CiCQE1 and CiCQE3 in 5-CQA remobilization *in planta*.

## 4 Discussion

Although enzymes capable of hydrolyzing 5-CQA have been identified in bacteria and fungi (Asther et al., 2005; Nieter et al., 2015; Negrel et al., 2016; Lo Verde et al., 2022), to date no such activity has been described in plants. Plant acyltransferases using 5-CQA as substrate have been shown to produce trace amounts of CA upon hydrolysis, but an efficient activity *in vitro* and *in vivo* has never been demonstrated (Lallemand et al., 2012; Miguel et al., 2020; Fu et al., 2021; Huang et al., 2024). In this study, we report the identification, cloning, biochemical characterization, and *in planta* functional analysis of two chicory esterases, CiCQE1 and CiCQE3, which hydrolyze 5-CQA and 3,5-DiCQA. Both enzymes show a high isomeric specificity toward 5-CQA, with minor activity on the isomers 3-CQA and 4-CQA. Regarding DiCQAs, CiCQE1 displays specificity for 3,5-DiCQA, whereas CiCQE3 is able to hydrolyze both 3,5-DiCQA and 1-5-DiCQA, although with lower efficiency. These caffeoyl-quinic acid esters are usually described as defense compounds against biotic and abiotic stresses in plants. Structurally, caffeoyl-shikimate and 5-CQA are closely related, both containing a caffeic acid moiety. The main difference lies in the presence of a double bond between C-1 and C-2 in shikimic acid and a hydroxyl group at C-1 in quinic acid (Lallemand et al., 2012). In higher plants, the release of CA from caffeoyl-shikimate, catalyzed by CSE, constitutes an important step in lignin biosynthesis (Vanholme et al., 2013). It has therefore been proposed that caffeoyl-quinic acids may act as storage forms of CA that can be remobilized (Silva et al., 2019). The first identification of *in planta* chlorogenate hydrolases in this study supports this hypothesis. Consistent with this role, both *CiCQE1* and *CiCQE3* are predominantly expressed in chicory roots, where 5-CQA and 3,5-DiCQA are the two majors accumulated phenylpropanoids. Moreover, none of them is able to metabolize caffeoyl-tartaric acids, which are structurally related compounds that are abundant in leaves but absent in roots of chicory. CiCQE1 and CiCQE3 show maximum activities at pH 4 and pH 6, depending on the buffer used. These pH optima are consistent with a functional localization of these enzymes within an acidic compartment, such as the vacuole, where caffeoyl-quinic acids are thought to be exported for long-term storage (Goupy et al., 1990; Li et al., 2019).

*In planta* experiment using transient expression in *N. benthamiana* confirmed the enzymatic activities observed *in vitro*. Expression of both CiCQE1 and CiCQE3 led to the accumulation of CA and caffeoyl-putrescine, which were absent in control leaves expressing an empty vector. Caffeoyl-putrescine is a phenolamide synthesized by BAHD acyltransferases using putrescine and caffeoyl-CoA as substrates (Peng et al., 2016a; Cao et al., 2024; Roumani et al., 2025). This compound accumulates in tobacco in response to biotic stress. Since control tobaccos were also infiltrated with agrobacteria, accumulation of this compound in response to pathogen attacks can be excluded in our experimental conditions. Therefore, the increased accumulation of *N*-caffeoyl-putrescine in leaves expressing CiCQE1 and CiCQE3 is likely due to the increased biodisponibility of caffeoyl-CoA due to accumulation of CA. This observation suggests that CA produced by CiCQE1 and CiCQE3 serves as a substrate for the endogenous 4-coumarate-CoA ligase (4CL) in tobacco leaves, leading to the production of caffeoyl-CoA. The accumulation of caffeoyl-putrescine is a phenotype notably observed in mutants impaired in the biosynthetic pathways of cell wall components that accumulate phenolic intermediates (Van Der Rest et al., 2006; Serra et al., 2010; Gaquerel et al., 2013). Several genes involved in the biosynthesis of specialized metabolites have been shown to be organized into biosynthetic gene clusters (BGCs), which promote their co-expression and their co-selection through generations (Nützmann and Osbourn, 2014; Smit and Lichman, 2022). Examples of physically linked gene clusters include the biosynthetic pathways of noscapine, avenacin and durrhin (Takos et al., 2011; Winzer et al., 2012; Li et al., 2021). In the phenylpropanoids pathway, three gene clusters involved in the synthesis of tomato’s phenolamides have recently been described (Cao et al., 2024, 2025). These clusters contain acyltransferases, notably alongside 4CL or CcoAOMT homologs, leading us to investigate whether CiCQEs form BGCs. However, examination of the genomic environment of CiCQE1 and CiCQE3 in the published chicory genome did not show the formation of such clusters (**Supplementary Figure S6**). This random distribution is common to genes in the phenylpropanoid pathway, which are not usually co-localized in the genome, despite being tightly co-regulated. Through the analysis of the sequenced chicory genome, we have identified 106 genes encoding GDSL proteins in chicory. This number is close to those reported for *A. thaliana, Oryza sativa* or *Zea mays* where 105, 114 and 103 genes encoding GDSL were predicted, respectively (Shen et al., 2022). This family is ubiquitous in land plants but occurs in small amounts in bryophytes and charophytes. The significant expansion of GDSL copies in *Selaginella moellendorffi*, which diverged shortly after the appearance of vascular tissue, suggests that these proteins are involved in the formation of cell wall components. In particular, members of the GDSL family have notably been shown to be involved in the formation of cutin, hemicellulose and suberin (Girard et al., 2012; Yeats et al., 2012; Zhang et al., 2017, 2019; Ursache et al., 2021). In our experimental setup in tobacco leaves, caffeoyl-putrescine was the only phenolics, along with CA, which was overaccumulated following CiCQEs expression. However, given the chemical differences between chicory roots and tobacco leaves, the caffeoyl-CoA generated by CiCQEs may be involved in distinct biosynthetic pathways depending on cellular microenvironment and substrate availability. Overexpression and knock-out of CiCQE1 and CiCQE3 are then needed to confirm the role of these caffeoyl-quinic hydrolases in chicory roots and to identify pathways and conditions in which 5-CQA remobilization is involved. In addition, subcellular localization studies are needed to confirm the predicted vacuolar localization of these enzymes. Finally, plant-derived chlorogenate hydrolasesoffer promising applications across both industrial and agricultural sectors. Caffeoyl-quinic esters have significant activities in human health that are strongly dependent on their caffeoyl groups (Islam et al., 2003). However, these compounds are only partially absorbed across the intestinal barrier, whereas CA is almost completely absorbed (Olthof et al., 2001). The release of CA from plant by-products rich in 5-CQA, such as apple pulp and coffee marc, has been achieved using fungal 5-CQA hydrolase and could serve as an efficient source of CA for human health (Asther et al., 2005). Another fungal esterase has also been used to produce coffee and apple juice with increased CA content (Siebert et al., 2018, 2019). From a food quality perspective, 5-CQA is generally considered to be a better substrate for polyphenol oxydases (PPOs) than caffeic acid, although substrate preference may vary among plant species (Tilley et al., 2023). The oxidation of 5-CQA by PPOs leads to the formation of reactive quinones, which can negatively impact both the nutritional and organoleptic properties of foods products, notably by inducing undesirable color changes such as browning and greening. In this context, CiCQEs represent a promising solution to reduce 5-CQA levels. This reduction limits the formation of reactive quinones, while promoting the accumulation of CA with interesting biological activities. In addition to 5-CQA, CiCQEs are also able to hydrolyze 3,5-DiCQA and some of its isomers, further enhancing CA release (**Supplementary Figure S5**). One practical application of CiCQEs can be seen during the processing of chicory roots into flour, where PPO-induced browning reduces the whiteness of the final product. By overexpressing CiCQEs in chicory roots or treating extracts with purified enzymes, it would be possible to mitigate this browning effect using an enzymes native to the host plant. Moreover, these enzymes remain active across a broad pH range (4-8), ensuring consistent activity despite pH fluctuations. A close homolog to CiCQEs, IbICS from sweet potato, which we identified in a previous study, exhibits high efficiency when expressed in yeast, along with high thermal stability (Miguel et al., 2020). CiCQEs could therefore be used in industrial bioreactors to treat various samples rich in 5-CQA. Similarly, a fungal chlorogenate hydrolase has recently been shown to prevent seed greening in sunflower by reducing endogenous 5-CQA content (Lo Verde et al., 2022; Pepra-Ameyaw et al., 2023), demonstrating the broad applicability of these enzymes. A study performed on coffee showed that treating coffee extract with a fungal chlorogenate hydrolase reduced the accumulation of chlorogenic acid lactones and the bitterness of the final product as assessed by a sensory panel (Kraehenbuehl et al., 2017). The newly discovered CiCQEs in plants could then be used to treat chicory and coffee, reducing their bitterness while preventing their oxidation.

In the context of plant defense, CA functions as a precursor in the phenylpropanoid pathway, contributing to the biosynthesis of several specialized metabolites that enhance resistance to biotic and abiotic stresses. These key pathways include phenolamides, rosmarinic acid, flavonoid conjugates and lignin (Vogt, 2010). All of these play essential roles in strengthening plant cell walls, responding to oxidative stress, and defending against pathogens. The introduction of CiCQEs into plants via genetic engineering increases the availability of CA, and its remobilization for specific defense pathways, as demonstrated in this study with tobacco. Thus, these enzymes are promising candidates for improving the resilience of important crop species to biotic and abiotic stresses (Muroi et al., 2009; Kaur et al., 2010).

The identification of plant chlorogenate hydrolases, such as CiCQE1 and CiCQE3, offers new insights into the catabolism and *in planta* remobilization of 5-CQA derivatives, which are widespread across the plant kingdom. These processes might have a potential role in plant development under specific growth conditions.

## Data Availability

The original contributions presented in the study are included in the article/**Supplementary Material**. Further inquiries can be directed to the corresponding author.
