# Supplementary material for: Identification of the first plant caffeoyl-quinate esterases in Cichorium intybus
Source: Front Plant Sci. 2025 Aug 20;16:1632036. doi: 10.3389/fpls.2025.1632036 (PMC12405362; doi:10.3389/fpls.2025.1632036)
Supplement: Supplementary file 1 [file DataSheet1.pdf]

## Table S1: Sequences of primers used in this study

| Primer sequence 5' to 3' | | Gene | Use |
|---|---|---|---|
| TTAACACCATTGCCTCGGCT | Fwd | *CiCQE1* | qRT-PCR |
| CGGTAGGTTTCCGGGAACAA | Rev | | |
| ACAATGCCGCCATGCAAATT | Fwd | *CiCQE2* | qRT-PCR |
| CATTCCGCCGACGTGTTAAC | Rev | | |
| CCCACCGGACGTTGTTCTAA | Fwd | *CiCQE3* | qRT-PCR |
| ACCTGCCACCGCATAATTCA | Rev | | |
| GTTGGGTGCGCATCTCTAAT | Fwd | *TIP41* | qRT-PCR |
| AGCTCCGGCAGCTTTTACTT | Rev | | |
| CATGGGCTCAGAAATCACCT | Fwd | *PP2AA2* | qRT-PCR |
| ATTGGTCAACGATGGGGATA | Rev | | |
| TGCTTACCCTAGTGCCTCTGA | Fwd | *PP2AA3* | qRT-PCR |
| TTCCCAAATTTGTAGCAGCA | Rev | | |
| TGCTTCCGCCATCTACTTTT | Fwd | *CLATH* | qRT-PCR |
| TCCCAAGTTCCTTTGTTTGC | Rev | | |
| ggggACAAGTTTGTACAAAAAAGCAGGCTccaccATGGCGATTTCACCAACTTTTCT | Fwd | *CiCQE1* | Cloning into pEAQ-HT-DEST3 |
| ggggACCACTTTGTACAAGAAAGCTGGGTcGAGGCTTACAAAAGGCTTTGA | Rev | | |
| ggggACAAGTTTGTACAAAAAAGCAGGCTccaccATGGCGTTTTCTTCCAGTTCT | Fwd | *CiCQE2* | Cloning into pEAQ-HT-DEST3 |
| ggggACCACTTTGTACAAGAAAGCTGGGTcGATAGAACTCGATAACCCTACTCG | Rev | | |
| ggggACAAGTTTGTACAAAAAAGCAGGCTccaccATGGCGAATTCATCAAACGTGT | Fwd | *CiCQE3* | Cloning into pEAQ-HT-DEST3 |
| ggggACCACTTTGTACAAGAAAGCTGGGTcGATAGAACTTGAGAATCCTCCT | Rev | | |
