# Supplementary figures and images for: Identification of the first plant caffeoyl-quinate esterases in Cichorium intybus
Source: Front Plant Sci. 2025 Aug 20;16:1632036. doi: 10.3389/fpls.2025.1632036 (PMC12405362; doi:10.3389/fpls.2025.1632036)

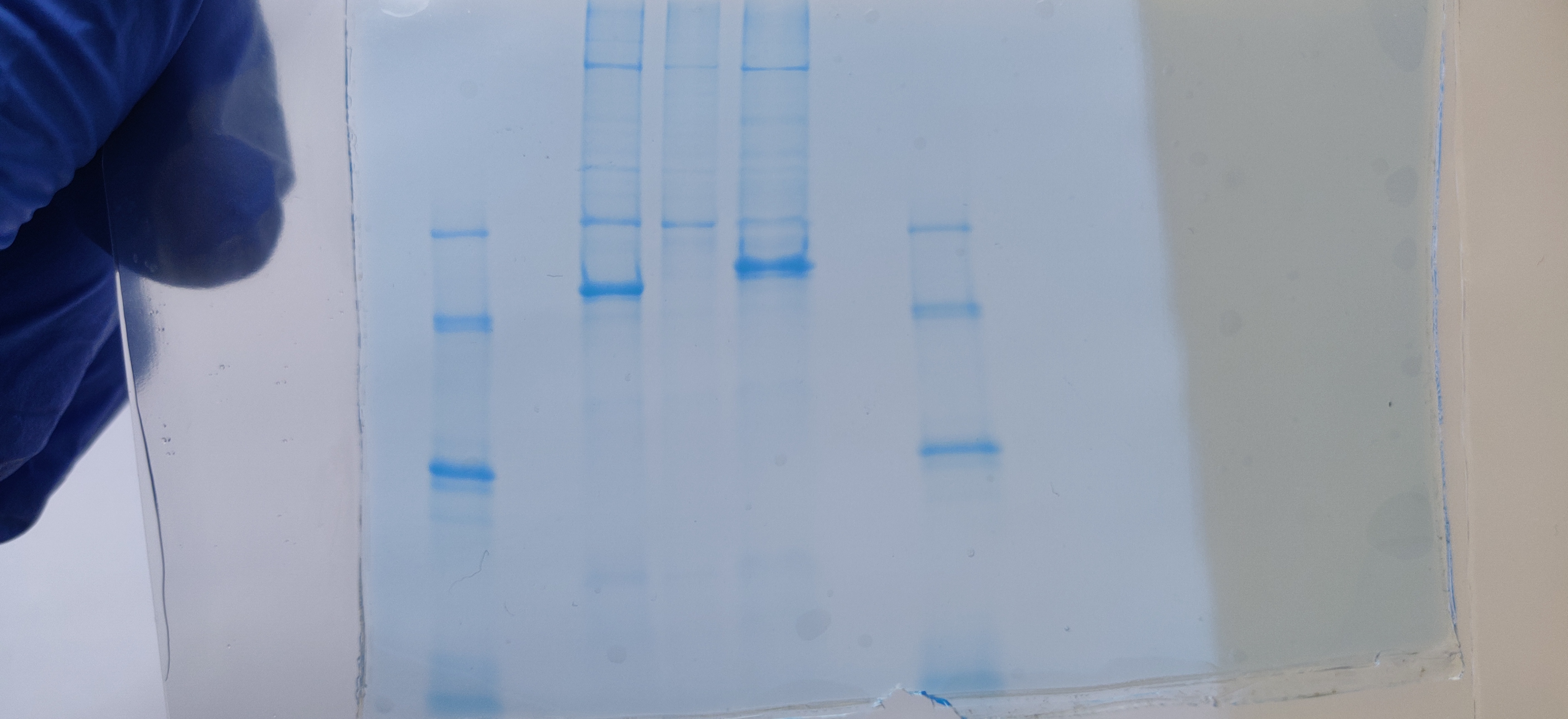

Supplement: Supplementary file 2 [file DataSheet2.zip › MALLAVERGNE_original_WB/Image 1.jpg]

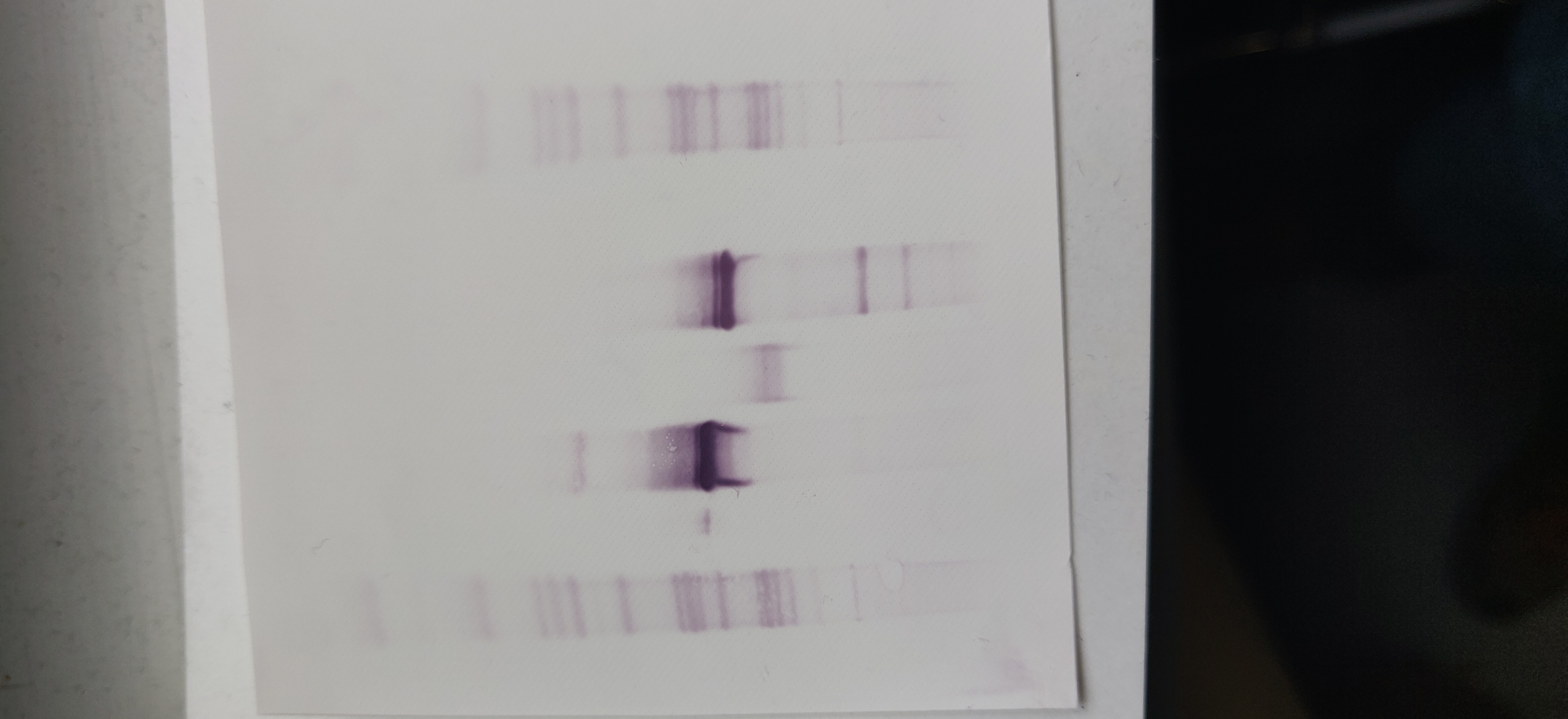

Supplement: Supplementary file 2 [file DataSheet2.zip › MALLAVERGNE_original_WB/Image 2.jpg]

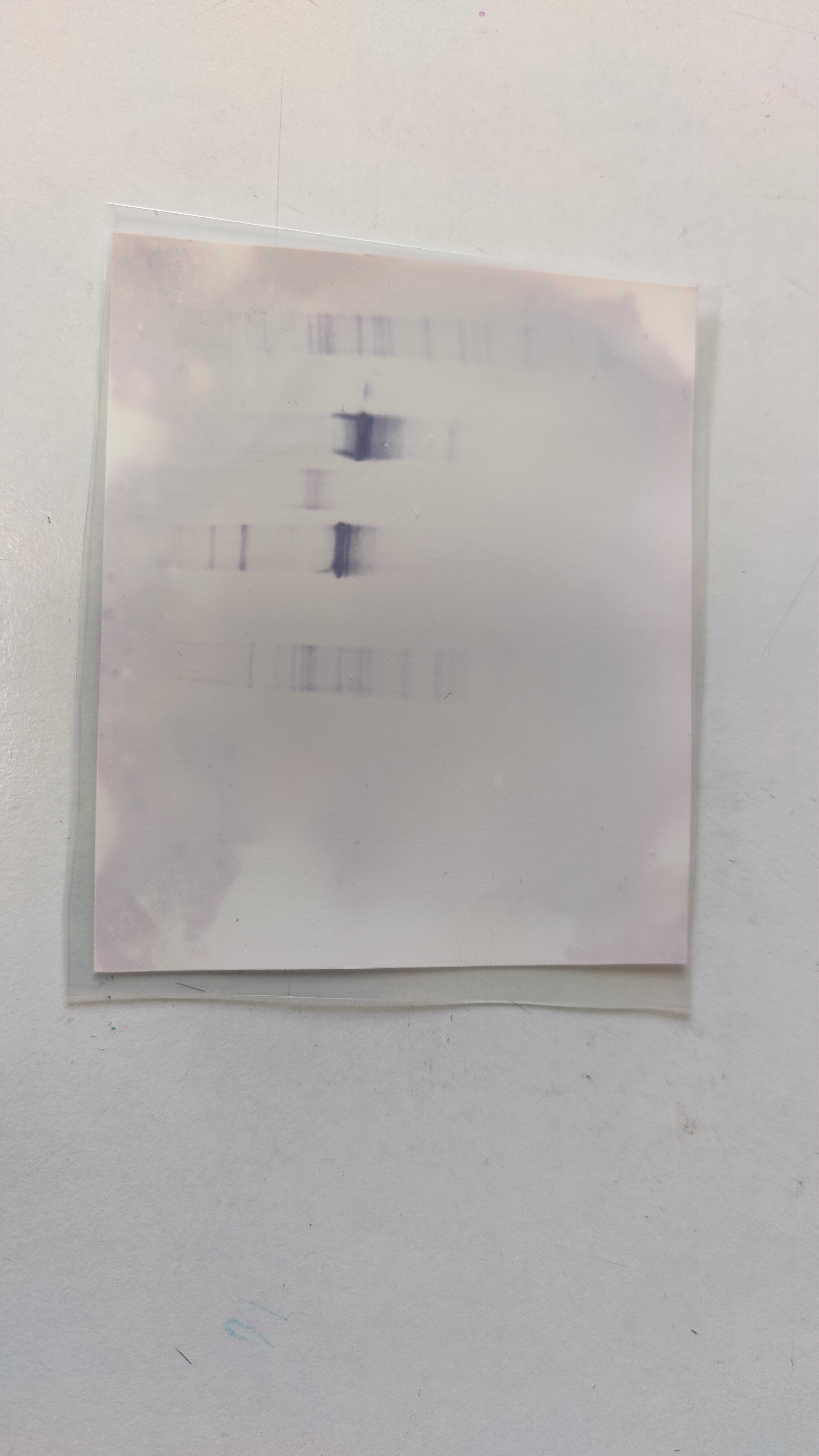

Supplement: Supplementary file 2 [file DataSheet2.zip › MALLAVERGNE_original_WB/Image 3.jpg]

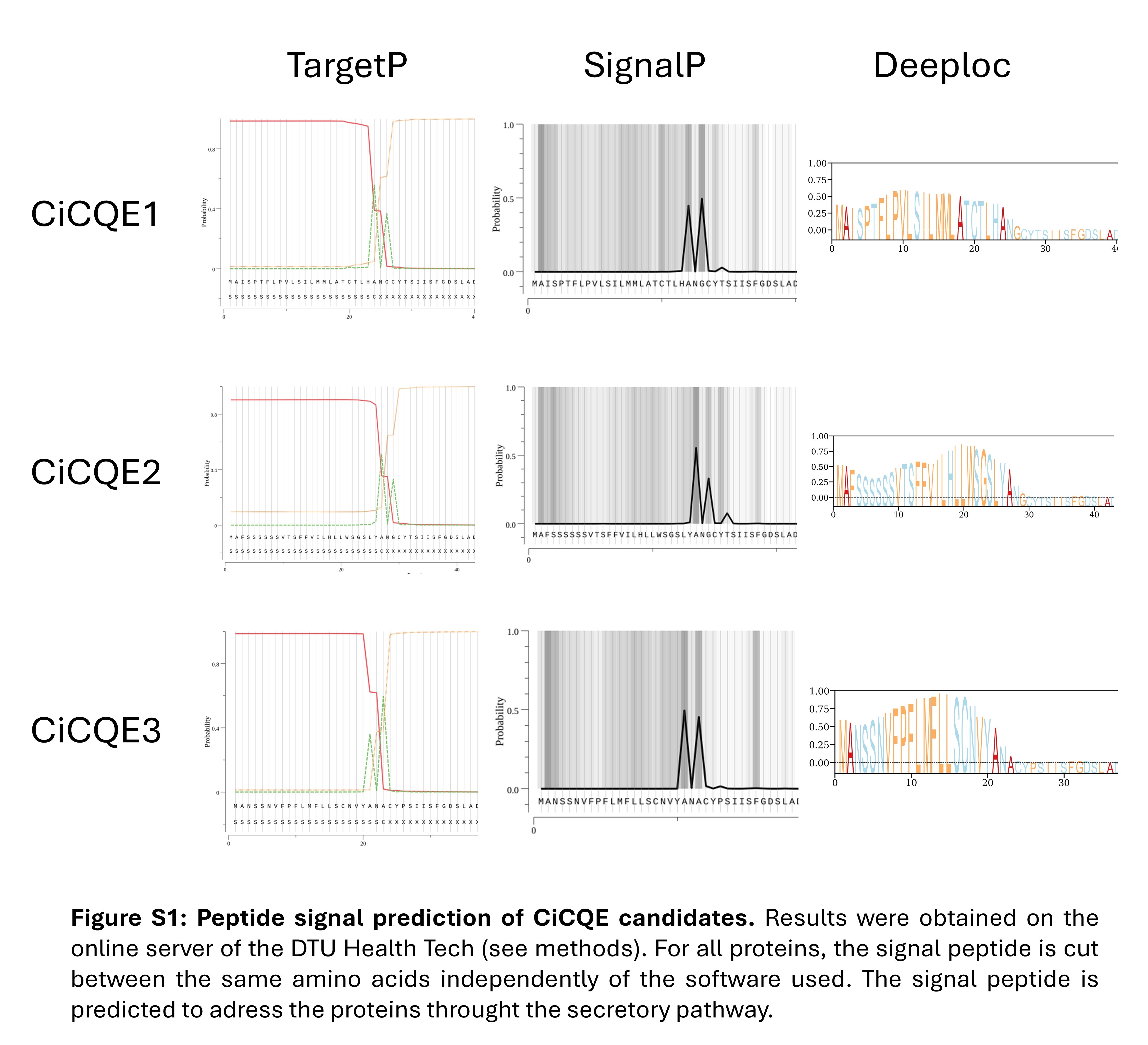

Supplement: Supplementary file 3 [file Image1.jpeg]

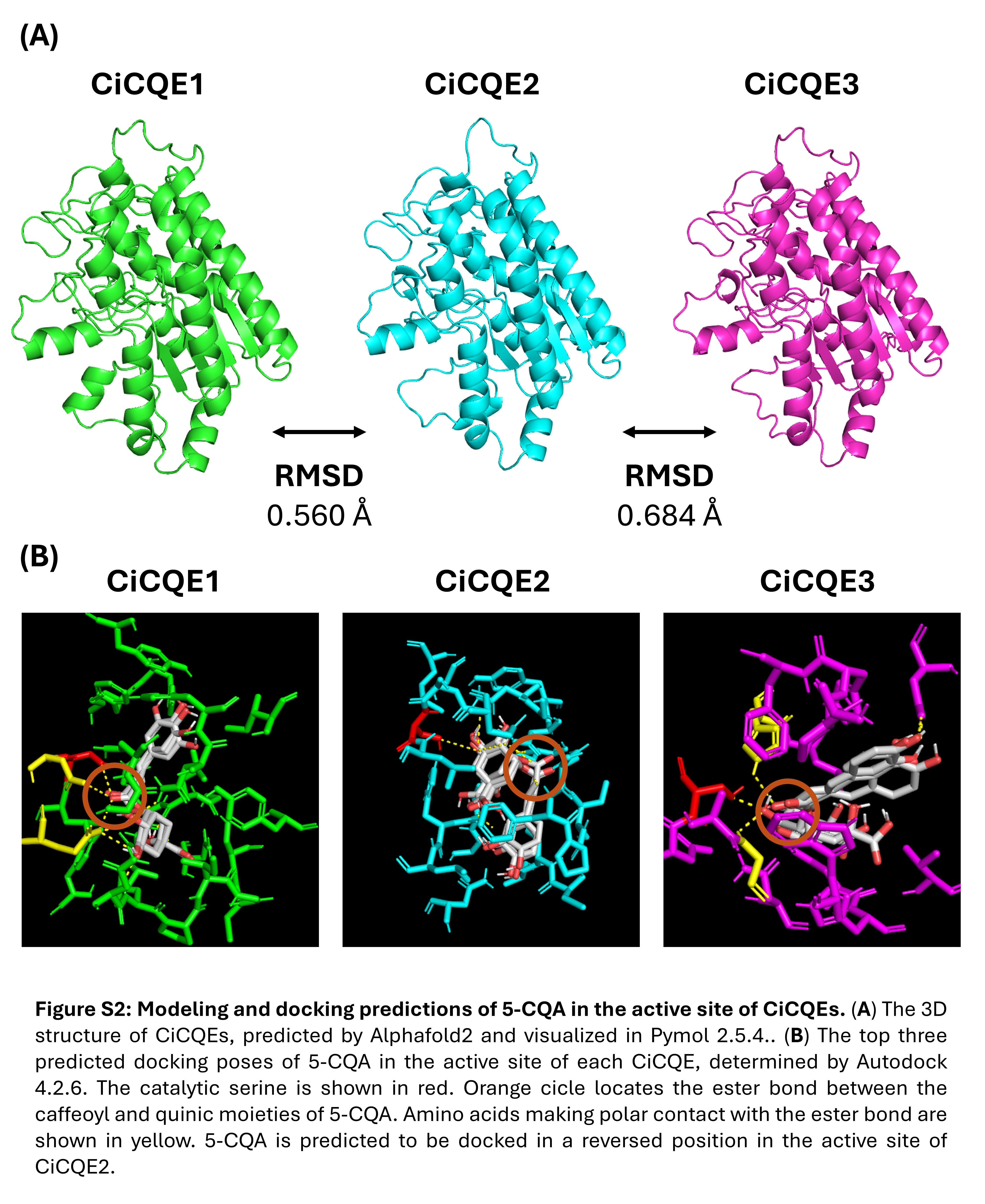

Supplement: Supplementary file 4 [file Image2.jpeg]

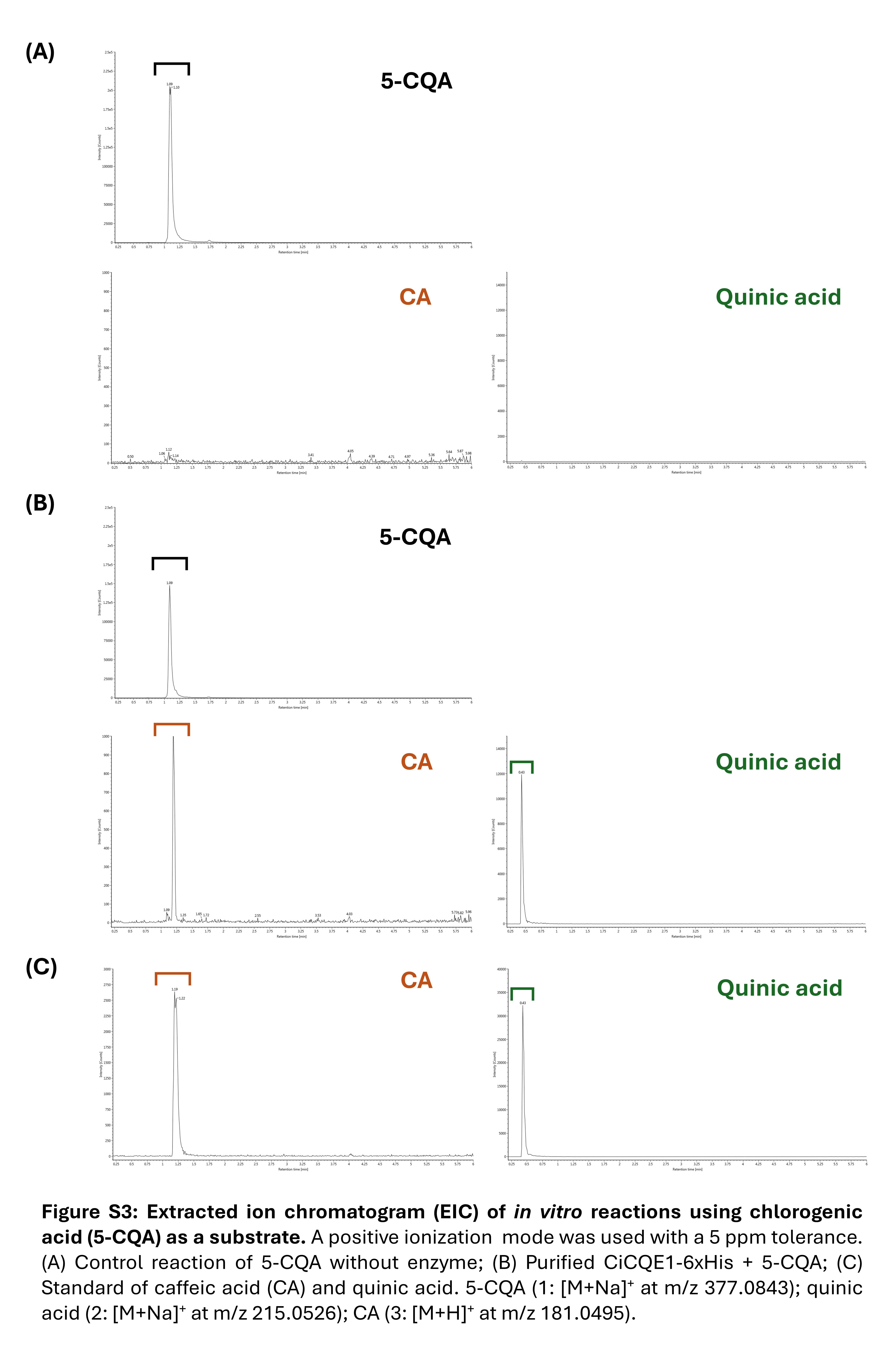

Supplement: Supplementary file 5 [file Image3.jpeg]

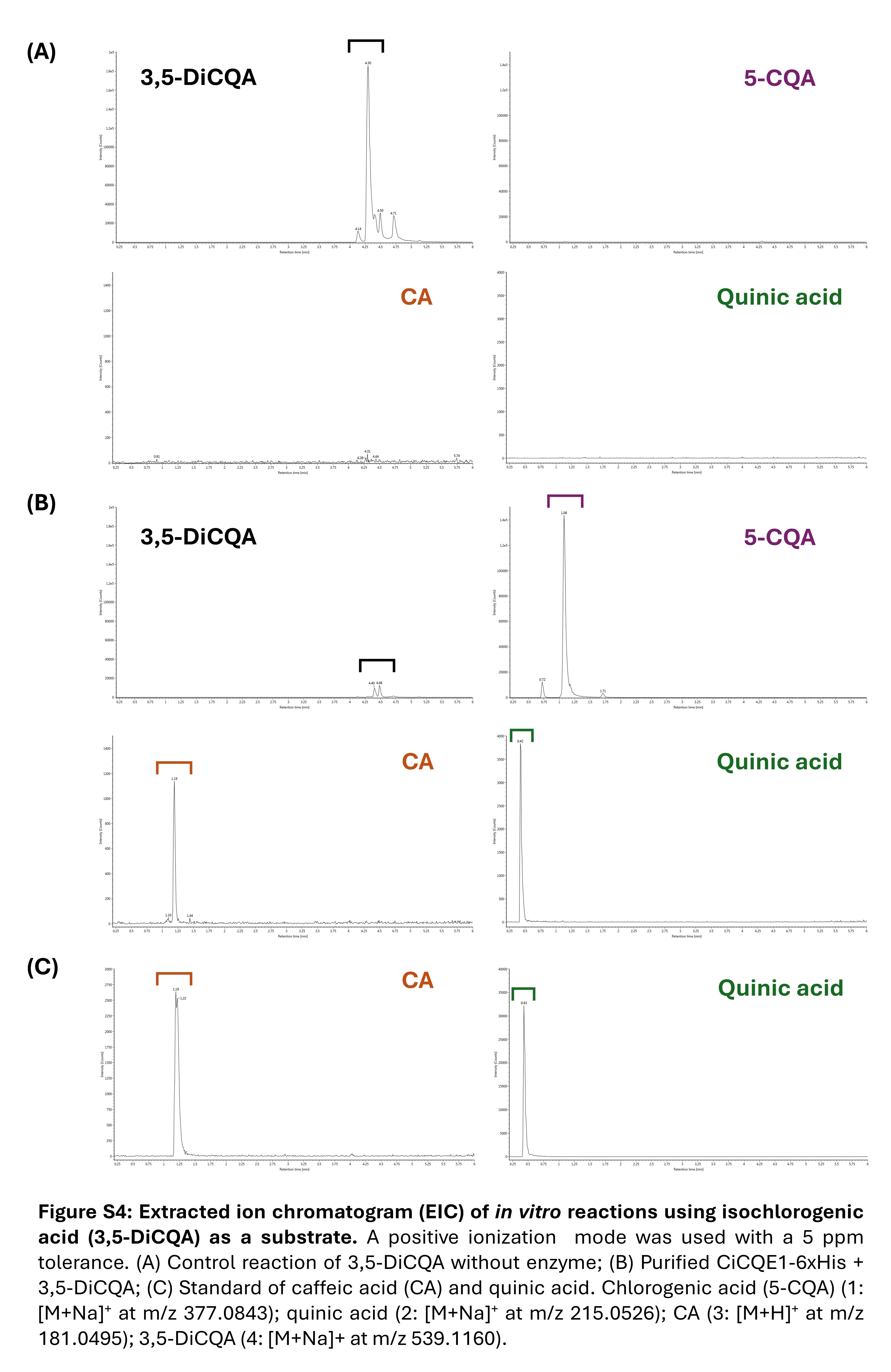

Supplement: Supplementary file 6 [file Image4.jpeg]

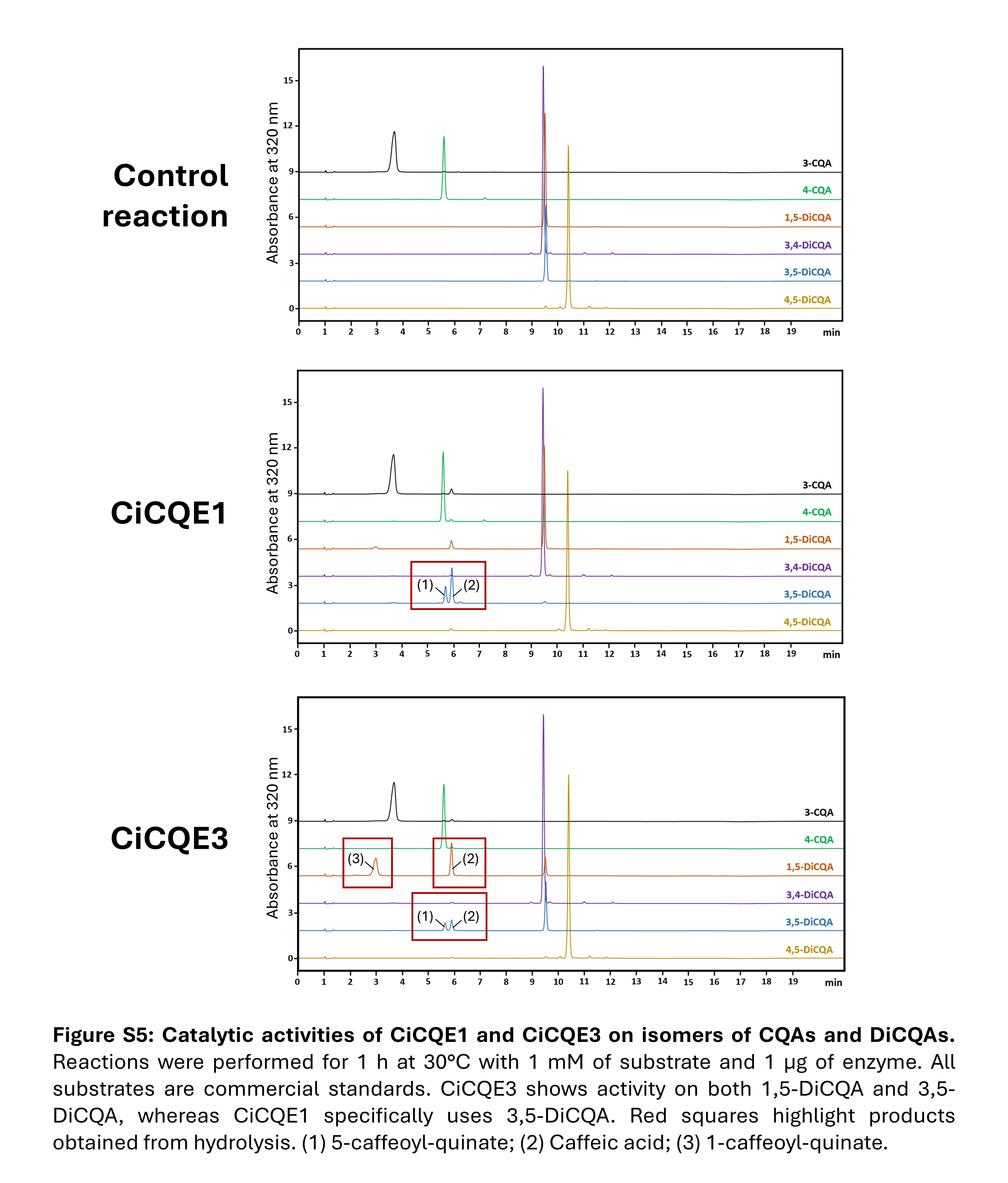

Supplement: Supplementary file 7 [file Image5.jpeg]

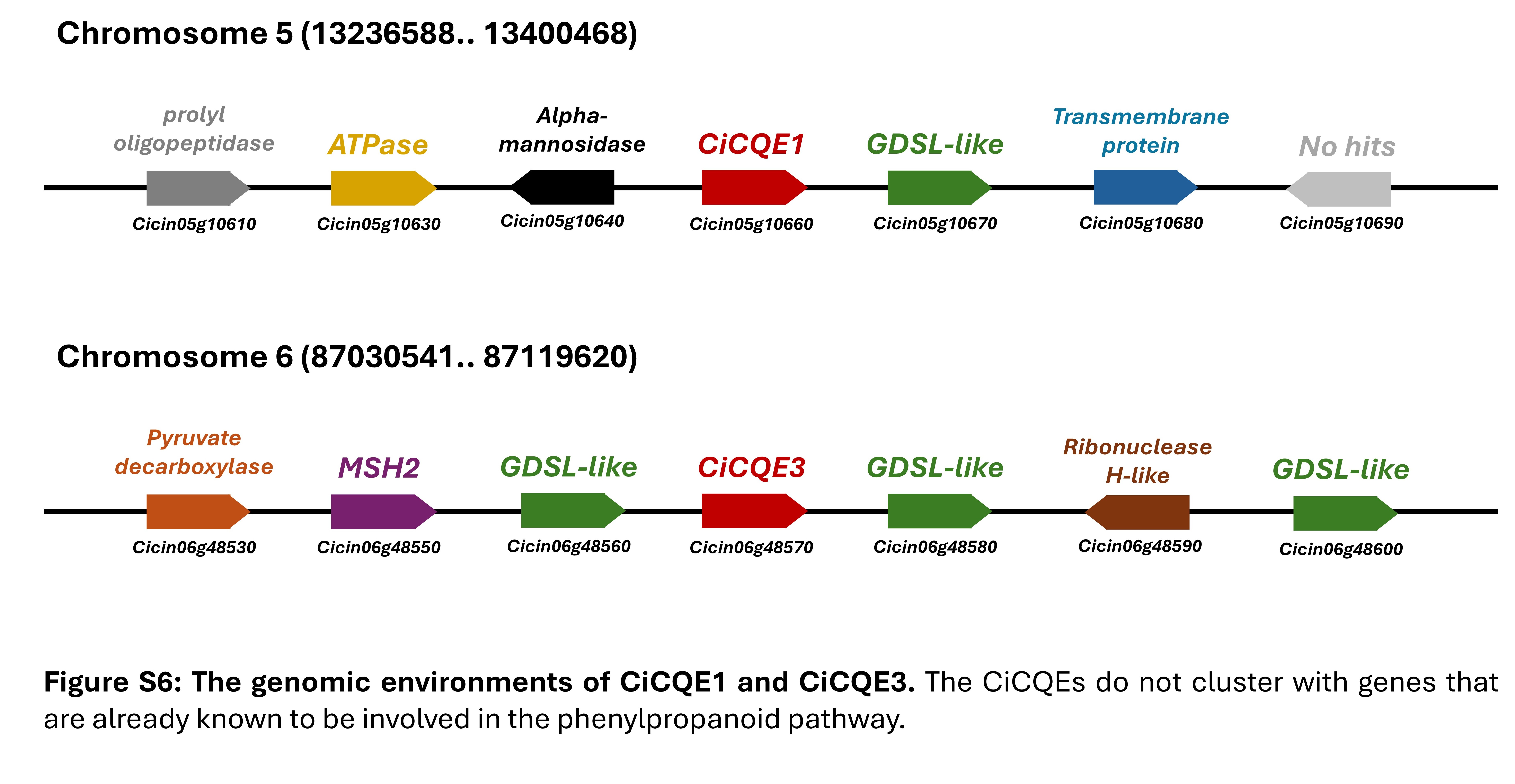

Supplement: Supplementary file 8 [file Image6.jpeg]
